# Association between socioeconomic status and dental caries in adolescents – a systematic review of cohort studies

**DOI:** 10.2340/aos.v85.46953

**Published:** 2026-10-07

**Authors:** Marit Slåttelid Skeie, Kristin Solveig Klock, Göran Dahllöf, Hedda Høvik, Ida Sofie Karlsen Sletten, Tone Natland Fagerhaug, Abhijit Sen

**Affiliations:** a Department of Clinical Dentistry, The Faculty of Medicine, University of Bergen, Bergen, Norway; b Center for Oral Health Services and Research, Mid-Norway (TkMidt), Trondheim, Norway; c Division of Orthodontics and Pediatric Dentistry, Department of Dental Medicine, Karolinska Institutet, Stockholm, Sweden; d Academic Librarian, The Medical Library, University of Bergen, Bergen, Norway; e Department of Public Health and Nursing, Norwegian University of Science and Technology (NTNU), Trondheim, Norway

**Keywords:** Adolescent, socioeconomic factors, dental caries, epidemiology

## Abstract

**Introduction:**

Early adolescence is a critical period for caries initiation, underlining the need for updated knowledge on effective caries indicators. This systematic review of cohort studies aimed to assess the association between socioeconomic status (SES), involving a wide range of indicators, and dental caries in adolescents aged 12–19.

**Materials and methods:**

Four databases (MEDLINE, Embase, Cochrane Library, and Web of Science) were systematically searched for relevant publications from January 1, 2000, through December 5, 2025. SES and SES proxy indicators were parental education, income, and occupation, and adolescents’ school type, their social capital, and immigrant background. Composite SES indicators combined the aforementioned variables. The risk of bias was evaluated using a modified Newcastle-Ottawa Scale.

**Results:**

Studies (*n* = 21) from 11 countries across five continents were included, covering approximately 66,000 adolescents. An inverse relationship was documented between caries and mothers’ education (six of seven studies), fathers’ education (three of four studies), and parental income (three of six studies). School type (three studies), social capital (two studies), immigrant background (four studies), and composite SES indicators (five of six studies) were also linked to caries. However, the effect measures and definitions of exposures and outcomes were not consistent.

**Conclusions:**

An inverse association was observed between maternal education and caries, while corresponding associations with fathers’ education and parental income were weaker and less consistent.

This type of association was also observed for adolescents’ school types, their social capital, and composite SES indicators. Immigrant background served as a caries predictor. However, the substantial heterogeneity in the included studies did not allow for meta-analyses and limited definite conclusions. The findings underscore the need to develop and validate robust composite SES measures applicable across diverse global contexts.

## Introduction

Socioeconomic health disparities have been a topic of interest for centuries [1]. Numerous studies have consistently reported a socioeconomic gradient in both general and oral health [2, 3]. Efforts in various welfare states have also been made to address this socioeconomic gradient in oral health [4]. Despite these initiatives, a global systematic review and meta-analysis of observational studies from 2000 to 2013 reported that low socioeconomic position (SEP) remained associated with a higher caries risk than high SEP, and that the SEP gradient in caries appeared especially strong in the most developed countries [5].

In the literature, the terms socioeconomic status (SES) and SEP are often used interchangeably. While SEP primarily refers to an individual’s employment status, occupation, or occupational class [6], SES is considered a more multidimensional measure that also includes education and income in addition to occupation. Nevertheless, the influence of SES or SEP on caries outcomes is not straightforward and is mediated by multiple factors. One such contextual mediating factor is the existing system for providing and utilizing oral healthcare [7]. Oral health-related attitudes exemplify individual attributes that affect this association. To better understand the complexity of how SES is linked to oral health, published theoretical explanations are useful. One such publication [8] has given four current explanations: the materialist explanation (factors beyond individual control), cultural/behavioral explanations (behaviors influenced by cultural norms of behavior), the psychosocial perspective (psychosocial level of stress), and the life course perspective (embodiment of prior living conditions). Another publication [9] gives a conceptual framework in which social and commercial determinants of oral health are combined. This framework highlights interacting influences and processes.

Socioeconomic disadvantage is particularly harmful when experienced early in life and is known to be a key risk factor for early childhood caries (ECC) [10]. However, adolescence marks a period of increased independence [11], and during this period, personal behavioral and dietary habits may influence the impact of SES on caries [12]. A lower association between SES and caries during early adolescence is reported [13], which may partly be explained by fewer teeth due to the exfoliation of primary molars or the lack of proximal contact in erupting permanent teeth. Another aspect is the longstanding practice in caries epidemiology of omitting enamel caries when measuring caries. This is problematic because enamel caries occurring between the ages of 12 and 15 accounts for half of the total caries experience [14], suggesting that without including enamel caries, the point estimates for the association between SES and caries may be biased.

Generally, systematic reviews and meta-analyses have, over the years, established solid evidence for a pronounced association between SES and caries. As caries is cumulative throughout life, analyzing this impact in a group of adults will not give a distinct description for adolescents [15]. Only a few systematic reviews have addressed this relationship by restricting the SES data to adolescents alone, without the influence of other age groups. In those reviews [16, 17], the scientific evidence supporting an inverse relationship was found to be relatively weak. Questions remain about the extent to which reported disparities in caries among adolescents are truly linked to SES, and whether commonly used parental SES indicators are appropriate. Traditional SES indicators have also been reported inconsistently related to adolescents’ health outcomes [18, 19]. The rationale for this systematic review is the uncertainties and missing knowledge in research literature about the SES-caries association during adolescence. Increasing knowledge in this field is important for selecting effective adolescent-focused promotive and preventive strategies.

In this systematic review of cohort studies, we therefore wanted to assess commonly used and other less commonly used SES indicators to examine their influence on caries in adolescents. The SES indicators included were parental education, adolescents’ type of school attended, and parental income and occupation. We also wanted to assess SES proxies such as adolescents’ social capital and immigrant background, and SES indicators that encompassed composite variables, consisting of different types. Only cohort design studies were selected because they are considered less susceptible to selection and recall bias and provide stronger evidence of temporal relationships.

The research question was: Is there an association between SES, across a range of indicators, and dental caries among adolescents aged 12–19 years?

## Materials and methods

This systematic review followed the Preferred Reporting Items for Systematic Reviews and Meta-Analyses (PRISMA) 2020 guidelines [20] and is registered in the international database of prospectively registered systematic reviews, PROSPERO 2024 (CRD42024542582). Amendments to the protocol included extending the search period and omitting meta-analysis. The decision not to perform meta-analysis was made post hoc because, generally, the number of eligible studies was too low, and a wide spectrum of heterogeneity prevented quantitative synthesis. The sources of heterogeneity were different types of SES indicators, inconsistencies in the defined caries outcome, and variability of effect measures used. Two previous attempts were made to do a meta-analysis. Effect of mother’s education on caries incidence with its associated funnel plot to assess publication bias are presented in Appendix 1. 1a and Appendix 1. 1b. Effect of parental income on caries incidence is shown by Appendix 1. 1c.. In these analyses, the concerns were about a too low number of studies and inconsistencies in caries outcome.

### Eligibility criteria

*Inclusion criteria* were publications in Scandinavian or English that reported a possible association between SES (the exposure variable) and caries incidence or caries increment in the permanent dentition of 12–19-year-olds. Studies in which SES was not the primary exposure were also included. The study design was limited to cohort studies (prospective or retrospective). If a study was published in multiple papers, the most recent, the one with the largest sample size, or the most relevant to this topic was selected. *Exclusion criteria* encompassed reviews, meta-analyses, theses, ecological studies, ‘grey’ literature (conference abstracts, dissertations, reports), and studies involving specific, non-representative populations such as socioeconomically deprived groups (including Indigenous or tribal populations), dental students, or groups with various medical conditions. Studies that relied solely on neighborhood or geographic information without additional SES evaluations, as well as intervention studies that could influence the described association, were also excluded. Finally, we excluded studies that used SES estimates based on self-estimation (e.g. satisfaction with their SES or their perception of SES) or self-reported caries status. Appendix 1. 2 outlines a table for exclusion criteria, while the potential impacts of these exclusions on the results are evaluated in the discussion section.

### Information sources

Studies published in peer-reviewed journals from January 1, 2000, until the final search on December 5, 2025, were retrieved from four databases (MEDLINE, Embase, Cochrane Library, and Web of Science) with the assistance of an academic librarian (ISKS) and peer-reviewed by another academic librarian. Furthermore, hand searches were conducted for publications in Scandinavian (Norwegian, Swedish, or Danish) dental journals and by using the reference lists of all included publications and other systematic reviews related to the topic.

### Search strategy

The search terms are presented in Appendix 1. 3. The publications were imported into the Rayyan Systematic Review Software [21], but the AI component was not used. Duplicates were removed using Bramer’s method [22] in the EndNote reference manager.

### Selection process

One reviewer (MSS) screened all publications by title and abstract (phase 1) three times, while another reviewer (KSK) screened once, covering 10% of the publications (every tenth publication). The reviewers’ selection agreement was 97.3%, and any disagreement was resolved through a consensus decision. In the further screening process (phase 2), both reviewers independently read the full text of the publications marked with the ‘Maybe’ button in Rayyan.ai. This functions as an intermediate decision between ‘Included’ and ‘Excluded’. Potentially eligible studies were re-examined, and when uncertainty remained, a third reviewer (AS) assessed the full text and contributed to the final inclusion decision.

### Data collection process

Three reviewers (MSS, AS, KSK) collaborated to extract the following data from the included studies: study ID, first author’s name, publication year, country and its Human Development Index (HDI) [23], cohort study type, study name or description, study period, follow-up duration, baseline sample size, child age at baseline, number of participants at follow-up, caries diagnostic method, use of bitewing (BW) radiographs, whether enamel caries were included, examiner calibration, number of examiners, exposure(s), outcomes (caries incidence/mean caries increment), and confounders.

### Data items

#### Outcome

The outcome was presented using the DMFT/S index (Decayed, Missing, and Filled Tooth/Surfaces) [24] to indicate either caries incidence (binary count of new caries cases) or the mean caries increment (increase in the number of caries-affected teeth/surfaces, ΔDMFT/ΔDMFS). The ‘D’ component of DMFT/S was defined either to include enamel caries (enamel level) or to be restricted to dentin caries (dentin level, excluding enamel caries). Caries risk was thus, over a specific period, defined as caries incidence or as caries increment. Additionally, caries severity was defined as an outcome when the caries increment exceeded a specified threshold.

#### The SES variable (exposure)

SES exposures included parental education, the adolescents’ type of school attended, and parental income and occupation, whereas SES proxies encompassed the adolescents’ social capital and immigrant background. SES exposures were also treated as composite variables, combining the aforementioned variables. Additionally, the HDI [23] of the respective country was evaluated based on the year the participants were first included in the study, classified as ‘Very high’, ‘High’, ‘Medium’, and ‘Low’.

#### Effect measures

Point estimates used to measure the association between SES and outcome were odds ratio (OR), incidence rate ratio (IRR), and relative risk (RR) with 95% confidence interval (CI). When beta (β) coefficients were applied, the standard error (SE) was reported. If the CIs were lacking, the authors of the study were contacted and asked if they could provide this information. Criteria for conducting meta-analyses included a minimum of three studies and consistent definitions of SES exposure variables and outcome measures.

### Study risk of bias assessment

To evaluate the quality of the included studies, modifications were made to the three categories (Selection, Comparability, Outcome) of the Newcastle-Ottawa Scale (NOS) [25] for cohort studies (https://www.ncbi.nlm.nih.gov/books/NBK115843/bin/appe-fm3.pdf). The modified NOS is presented in Appendix 1. 4 (stars in the standard NOS version are replaced by points). Total scores could range from 0 to 10 points, categorized as low (0–3), medium (greater than 3–6), and high (greater than 6–10) study quality. For caries at the dentin level, the decision regarding the question, ‘Was the follow-up long enough for outcomes to occur?’ was set at a minimum of 4 years of follow-up for 10–11-year-olds and up to 17 years of age, and at more than 7 years of follow-up for older participants. These limits were based on research on the median time for caries progression by Mejàre et al. [26]. No limit was set for enamel lesions. In the modified NOS, a study could be awarded two points in the Outcome category instead of one. This was done because in caries epidemiology, the caries assessment is crucial and especially relevant for study quality. As this part in the standard NOS (a, b) was not suitable for this study, the text was changed to a) calibration of examiners (0.5 point), and with reliability caries tests (0.5 point), b) use of BW radiographs (1 point). In the Selection category, part 3, ‘Ascertainment of the exposure’, the text in the standard NOS (a and b) was modified to a) standardized record/use of known measurements (1 point), b) validated questionnaires/structured interviews (1 point). Lastly, in the category ‘Comparability’, the text was altered from standard NOS to 0.25 points for each confounder. This was done because in the standard NOS, mentioning adjustment for only age and sex would qualify for the maximum value, 2 points. Two reviewers (MSS, KSK) independently assessed each included study.

## Results

### Study selection

Figure 1, the flow diagram based on the PRISMA 2020 statement [20], outlines the results of the search and selection process. Initially, 2817 publications were identified (MEDLINE: 850, Embase: 1016, Cochrane Library: 348, Web of Science: 603). After excluding publications based on title and/or abstract and publications not written in English or Scandinavian languages (*n* = 31), 80 publications were considered potentially relevant, of which 5 were identified from reference lists. After the exclusions described in detail in Appendix 1. 5, 34 publications remained. Of these, 13 publications were excluded because they reported the same studies as included publications. The respective publications and the reasons for selecting them are presented in Appendix 1. 6.

**Figure 1:**
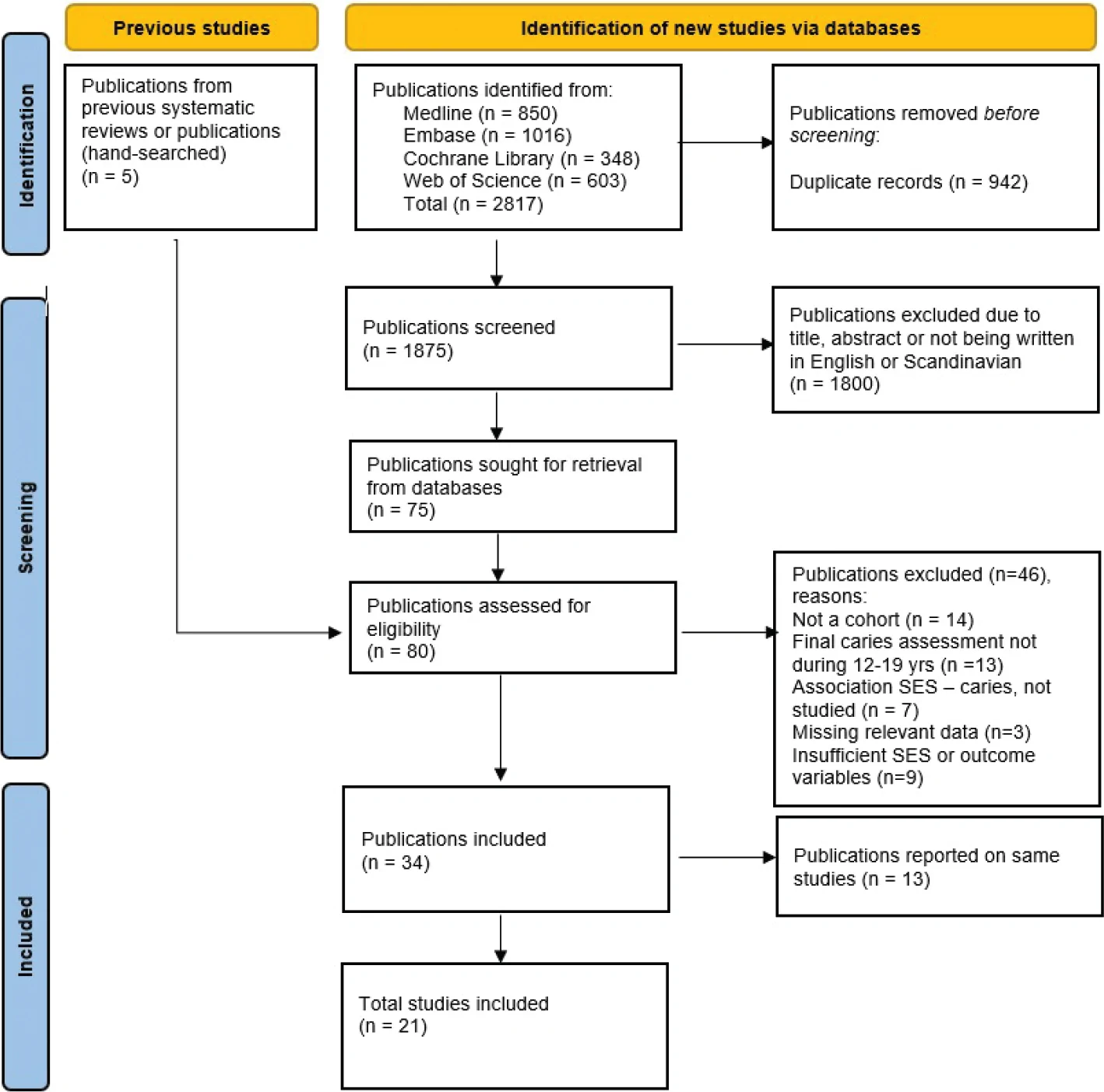
Flow chart based on the PRISMA 2020 statement.

### Study characteristics

As shown in Table 1, the 21 cohort studies (18 prospective and three retrospective) included data from approximately 66,000 adolescents aged 12–19 years, representing 11 countries across five continents (Asia, North America, South America, Europe, and Oceania). Most studies were published between 2010 and 2019 (*n* = 10), and between 2020 and the final search date, December 5, 2025 (*n* = 8). The majority of studies were from Brazil (*n* = 6) and Sweden (*n* = 4). Using HDI [23], 13 studies reported a very high HDI and five a high HDI, while three reported a medium HDI. Studies on caries incidence and increment were reported more frequently at the tooth (T) level (*n* = 14) than at the surface (S) level (*n* = 7). Ten studies used the World Health Organization (WHO) caries diagnostic criteria [27], but five- and six-grade/code caries diagnostic systems were also applied. However, only three studies included enamel caries in analyses of the association between SES and caries. Calibration of the examiners was performed in 17 studies, although not all provided results from caries reliability tests. Seven studies reported using BW radiographs. Additionally, the duration of the follow-up periods varied.

**Table 1a:** Characteristics of included cohort studies in the systematic review.

ID	First author Publication year Country The Human Development Index (HDI): Year-HDI (very high, high, medium, low)	Type of cohort study name or description	Study period Follow-up duration	Sample size at baseline Child’s age at baseline	No. of participants at follow-up for caries examination	Caries diagnostic method Bitewings (BW): Yes, No, Not reported (NR)	Enamel caries (included/not included
1	Alm A et al. [28] 2012 Sweden Very high HDI 1988 – 0.891	Prospective Study from the Municipality of Jönköping	1988–2002, 14 years follow-up	671: 1-year-old (plaque examined) 555; 3-year-olds 517: 6-year-olds	539 555 517	Socialstyrelsen, 1988 BW: Yes	Included
2	Bernabé E et al. [29] 2011 UK Very high HDI 2001 – 0.869	Prospective East London Adolescent Community Health Survey (RELACHS)	2001–2005, 4 years follow-up	689; 11–12-year-olds	689 (boys 318, girls 371)	WHO criteria BW: No	Not included
3	Brondani B et al. [30] 2024 Brazil High HDI 2010 – 0.722	Prospective Children at primary health care units at the National Children’s Vaccination Day. South Santa Maria, Brazil	2010–2020 10-year follow-up	639, 1–5-year-olds 449, 8–12-year-olds	429 (boys 220, girls 209)	Ismail et al. 2007). ICDAS codes 3–6 BW: NR	Not included
4	Costa, LR et al. [31] 2023 Brazil High HDI 2016 – 0.753	Prospective Study from Urban region of Manaus, Brazil	2016–2017, 1-year follow-up	415; 12-year-olds	312 (boys 136, girls 176)	WHO criteria BW: NR	Not included
5	Gerdin, EW et al. [32] 2008 Sweden Very high HDI 1997 – 0.878	Prospective Data from County Council registers	1997–2003, 6 years follow-up	2303; 6-year-olds 4305: 10-year-olds	2303 (boys and girls- NR)	Socialstyrelsen, 1988 BW: Yes	Not included
6	Hall-Scullin E et al. [33] 2017 England Very high HDI 2006 – 0.894	Prospective Class 3 school pupils; East Lancashire in the northwest of England	2006–2013, 7 years follow-up	5048, 7–9-year-olds	5048 (boys and girls- NR)	UK national diagnostic protocol (Pine et al. 1997) BW: NR	Not included
7	Julihn A et al. [34] 2010 Sweden Very high HDI 2000 – 0.903	Retrospective Data from Public Dental Health Service, private practitioners, Pediatric Dentistry (Karolinska Institutet)	2000–2007, 6 years follow-up	15,538; 13-year-olds	15,538 (boys 7810, girls 1728)	Diagnostic method *(described in the publication)* BW: Yes	Not included
8	Jung SH et al. [3] 2018 South Korea Very high HDI 2011 – 0.895	Prospective Adolescents from high schools (urban and rural) of Gangneung, South Korea	2011–2013, 2 years follow-up	1267; 15-year-olds	1073 (boys 566, girls 507)	Korean National Oral Health Survey 2010 as standard BW: NR	Not included
9	Källestål C et al. [35] 2007 Sweden Very high HDI 1995 – 0.859	Prospective Nationwide Swedish public dental health clinics	1995–1999, 4 years follow-up	3373; 12-year-olds 3109, 14-year-olds	2845 (boys and girls: NR)	Källestål C 2000 (described) BW: Yes	1) Not included 2) Included
10	Llena C et al. [36] 2020 Spain Very high HDI 2005 – 0.851	Retrospective Children born in 2005, enrolled in a Community Dental Program, Valencia, Spain	2005–2018, 7 years follow-up	278, 5-year-olds	278 (boys 137, girls 141)	Ismail et al. 2007 A: ICDAS codes 3–6 B: ICDAS codes 1–6 BW: NR	1) Not included 2) Included
11	Lu HX et al. [37] 2011 China Medium HDI 2001 – 0.597	Prospective Hong Kong Oral Health Survey from 2001	2001–2007, 6 years follow-up	638, 12-year-olds	324 (boys and girls NR)	WHO criteria BW: NR	Not included
12	Ortiz FR et al. [38] 2022 Brazil High HDI 2012 – 0.732	Prospective School children from Porto Alegre, South Brazil	2012–2018, 6 years follow-up	1134, 12-year-olds	768 (boys 341, girls 427)	WHO criteria BW: NR	Not included
13	Page LA & Thomson WM [39] 2011 New Zealand Very high HDI 2003 – 0.908	Prospective Taranaki adolescents from New Zealand	2003–2006, 3 years follow-up	430, 13-year-olds	255 (boys 127, girls 128)	WHO criteria BW: NR	Not included
14	Peres MA et al. [40]. 2018 Brazil Medium HDI 1999 – 0.663	Prospective Substudy of Pelotas study from 1993	1999–2009, 12 years follow-up:	359; 6-year-olds 339, 12-year-olds	307 (boys and girls: NR)	WHO criteria BW: NR	Not included
15	Pilecco RO et al. [41] 2020 Brazil High HDI 2012 – 0.732 High HD	Prospective Public schools in Santa Maria, Brazil	2012–2014, 2-year follow-up	1134, 12-year-olds	743 (boys 352, girls 391)	WHO criteria BW: NR	Not included
16	Saethre-Sundli HB et al. [42] 2020 Norway Very high HDI 2007 – 0.936	Prospective Norwegian school children from Akershus County	2007–2014, 7 years follow-up	5623, 5-year-olds	5623 (boys 1696, girls 1586)	Amarante et al. 1998 BW: Yes	Not included
17	Schmoeckel J et al. [43] 2015 Germany Very high HDI 1995 – 0.858	Prospective German schoolchildren from Greifswald	1994–2005, 10-year follow-up	521; 5-year-olds	170 (boys 83, girls 87)	WHO criteria BW: NR	Not included
18	Tagliaferro EP et al. [44] 2006 Brazil Medium HDI 1997 – 0.645	Prospective Brazilian schoolchildren, from Piracicaba, SP	1997–2004, 7 years follow-up	480, 6–8-year-olds Mean age: 7.1 years	206 (boys 93, girls 113)	WHO criteria BW: NR	Not included
19	Tan SHX et al. [45] 2021 Singapore Very high HDI 2009 – 0.910	Retrospective Singapore Dental Council	2009–2017, 8-year follow-up	29,617, 7-year-olds	29,617 (boys 15,049, girls 14,568)	Standard of School Dental Service BW: NR	Not included
20	Wang K et al. [46] 2020 China High HDI 2018 – 0.758	Prospective Study from Foshan city in Guangdong Province in southern China	2018–2019, 1-year follow-up	1016, 13-year-olds	1016 (boys, girls: NR)	ICDAS A: ICDAS codes 4–6 B : ICDAS codes 1–6 BW: NR	Not included
21	Warren JJ et al. [47] 2017 US Very high HD 1992 – 0.880/1995 – 0.886	Prospective Birth cohort study from Iowa Fluoride Study (IFS)	1992/95–2009, 4-year follow-up: From 5, 9, 13 years to 17 years.	396, 5-, 9-, 13-year-olds	396 (boys 183, girls 213)	Warren 2002 (described) BW: NR	Not included

ICDAS: International Caries Detection and Assessment System;

Ismail et al. (2007). The International Caries Detection and Assessment System (ICDAS). A six-grade/code caries diagnostic system.

Pine et al. (1997). Using a UK national diagnostic protocol, the threshold for caries was frank cavitation into dentin (D3).

Amarante et al. (1998). A five-graded scale, enamel (initial) lesions (1 and 2), dentin lesions (3-5).

Socialstyrelsen (1988). Initial caries can be assessed (D1), but was not assessed in present included studies.

Korean National Oral Health Survey (2010). Untreated caries, “D” component, was the only focus in this diagnostic tool.

Källestål (2000). Four scores (1-4) denoted caries. When enamel caries was clinically detected, the lesion was judged to extend into dentin.

Warren (2002). Cavitated lesions (grades 2-3) were distinguished from noncavitated lesions (grade 1).

**Table 1b:** Characteristics of included cohort studies in the systematic review.

ID	First author Publication year Country The Human Development Index (HDI): Year-HDI (very high, high, medium, low)	Calibration (Yes/No) No. of examiners at baseline and follow-up	Exposure (s)	Outcome Caries incidence (binary new caries cases) DMFT/S: Decayed, Missing, FilledTooth/Surfaces	Outcome Mean caries increment Increase in DMFT/S: (Δ DMFT/S)	Confounders
1	Alm A et al. [28] 2012 Sweden Very high HDI 1988 – 0.891	Yes *Examiners* Baseline: 1 Follow-up: 1		DFprox**S** ≥ 4		dfs at 6 years, consumption of sweets at 1 year, mother’s self-estimation of her oral health care.
			Immigrant background Both parents born in Sweden	OR (95% CI) 1.0 (ref)		
			Only father born abroad	1.5 (1.0–2.3)		
			Only mother born abroad	2.4 (1.0–5.5)		
			Both parents born abroad	3.7 (1.1–12.8)		
2	Bernabé E et al. [29] 2011 UK Very high HDI 2001 – 0.869	Yes *Examiners* Baseline: 2 Follow-up: 2 (same)	Multidimensional Scale of Perceived Social Support (MSPSS)		DMF**T-** Second permanent molar (newly erupted)	Sex, age, ethnicity, parental employment, dental behaviors.
			Total score		RR (95% CI) 1.00 (ref)	
			Family factor score Friends factor score Special factor score		0.98 (0.83–1.10) 0.80 (0.71–1.11) 0.81 (0.69–0.96)	
3	Brondani B et al. [30] 2024 Brazil High HDI 2010 – 0.722	Yes *Examiners* Baseline: NR Follow-up: NR			DM**T**	Visits to neighbors, dental attendance, household income, sex, skin color, SES context.
			Composite SES			
			Monthly household income: 4 quartiles (low to high) Parental education: < 8 years ≥ 8 years Household crowding 4 quartiles (low to high)		β: –0.28 (SE: 0.10)	
4	Costa, LR et al. [31] 2023 Brazil High HDI 2016 – 0.753	Yes *Examiners* Baseline: 5 Follow-up: 5 (same)	Social support		ΔDMF**T**	Sense of coherence, SES (representing number of goods, house crowding, parents’ schooling, family income).
			Friends Relatives Teachers Others		β: –0.026 (Bootstrap SE: 0.015)	
			Composite SES Durable goods, Overcrowding, Parental education, Monthly family income ≤ ½ BMW (Brazilian minimal wages). ½ to 1 BMW > 1 BMW		β: –0.046 (Bootstrap SE: 0.023)	
5	Gerdin, EW et al. [32] 2008 Sweden Very high HDI 1997 – 0.878	Yes *Examiners* Baseline: NR Follow-up: NR	Composite SES		DF**T**	Gender, childhood Body Mass Index.
			Socioeconomic clusters (based on available income, social allowances and parental education) I (high), II, III, IV, V (low)		Cluster V (low): 1.1 Cluster I (high): 0.6. *p* < 0.002	
6	Hall-Scullin E et al. [33] 2017 England Very high HDI 2006 – 0.894	Yes *Examiners* Baseline: NR Follow-up: NR	Composite SES		DMF**T**	Age, dmft, gender, ethnicity.
			Index of Material Deprivation quintiles IMDQ 1 (least deprived) IMDQ 2 IMDQ 3 IMDQ 4 IMDQ 5 (most deprived)		IRR (95% CI) 1.00 (ref)	
					1.06 (0.83–1.16) 1.34 (1.04–1.72) 1.39 (1.10–1.77) 1.77 (1.41–2.23)	
7	Julihn A et al. [34] 2010 Sweden Very high HDI 2000 – 0.903	NR *Examiners* Baseline: NR Follow-up: NR		ΔDMF**S**prox > 0		DMFT at 13 years, gender, parental education level, father’s country/region of birth, social welfare allowance (family), low-income range, no. of children in the family.
			Immigrant background Sweden Western Europe Eastern Europe Asia Africa South America	OR (95% CI) 1.00 1.47 (0.98–2.21) 1.44 (1.12–1.85) 1.15 (0.93–1.41) 1.33 (0.91–1.93) 0.93 (0.67–1.28)		
			Mother’s education Low ≤ 9 years Medium (10–12 years) High: > 12 years	OR (95% CI) 1.00 (ref) 0.91 (0.84–1.02) 0.73 (0.65–0.81)		
			Father’s education Low ≤ 9 year Medium (10–12 years) High: > 12 year	OR (95% CI) 1.00 (ref) 0.92 (0.82–1.00) 0.85 (0.77–0.95)		
			Parental income: High income range: > 253,000 SEK Low income: ≤ 253,000 SEK	OR (95% CI) 1.00 (ref) 1.15 (1.05–1.25)		
8	Jung SH et al. [3] 2018 South Korea Very high HDI 2011 – 0.895	Yes *Examiners* *Baseline: 1* Follow-up: 1 (same)		ΔD**T** > 0		Gender, perceived economic status, oral health habits, smoking experience, annual visits to the dental clinics, school type, parental education.
			School type General Vocational	OR (95% CI) 1.00 (ref) 2.68 (1.46–4.92)		
			Mother’s education High: College or above Low: High school or below	OR (95% CI) 1.00 (ref) 0.86 (0.43–1.73)		
			Father’s education High: College or above Low: High school or below	OR (95% CI) 1.00 (ref) 2.04 (1.05–3.98)		
			Composite SES Family affluence scale (0–9) High: > 5 Low: ≤ 5	OR (95% CI) 1.00 (ref) 1.80 (0.88–3.64)		
9	Källestål C et al. [35] 2007 Sweden Very high HDI 1995 – 0.859	Yes *Examiners* Baseline: 26 Follow-up: 23 (same) and 3 were new		ΔDMF**S** *Enamel caries not included*		Sex, ethnicity, sealants, eating candy, toothbrushing intervals, earlier preventive programs, water fluoridation.
			Occupation Civil servant Business Workers	RR (95% CI) 1.00 (ref) 1.02 (0.99–1.06) 1.04 (1.02–1.07)		
			Immigrant background – child Sweden Nordic/Western Europe Eastern Europe Other	RR (95% CI) 1.00 (ref) 1.04 (0.96–1.14) 1.25 (1.12–1.40) 1.07 (1.01–1.14)		
				*Enamel caries included*		
			Occupation Civil servant Business Workers	RR (95% CI) 1.00 (ref) 1.03 (0.99–1:07) 1.06 (1.01–1.12)		
			Immigrant background-child Sweden Nordic/Western Eastern Europe Other	RR (95% CI) 1.00 (ref) 1.18 (0.97–1.45) 1.25 (1.03–1.50) 1.12 (1.01–1.25)		
10	Llena C et al. [36] 2020 Spain Very high HDI 2005 – 0.851	NR *Examiners* Baseline: 1 Follow-up: 1 (same)			DMF**T** *Enamel caries not included*	dft, MIH, cariogenic diet, toothbrushing frequency, no parental caries experience 6 months before inclusion.
			Mother’s education No education/primary Secondary/higher		IRR (95% CI) 1.00 (ref) 0.21 (–0.13 – 0.54)	
					*Enamel caries included*	
			Mother’s education No education/primary Secondary/higher		IRR (95% CI) 1.00 (ref) 0.37 (0.13–0.60)	
11	Lu HX et al. [37] 2011 China Medium HDI 2001 – 0.597	Yes *Examiners* Baseline: NR Follow-up: NR			DMF**T**	Utilization of dental services
			Parents’ education: Primary or below Junior high school Senior high school Matriculation or above		β coefficient: NR *p* > 0.05.	
			Monthly Household income: < HK$5001–HK$10,000 HK10,000–HK$20,000 HK$20,000–HK$30,000 HK$30,000–HK$40,000 > HK$40,000		β coefficient: NR *p* > 0.05	
12	Ortiz FR et al. [38] 2022 Brazil High HDI 2012 – 0.732	Yes *Examiners* Baseline: 4 Follow-up: 4		ΔDT > 0		Sex, visit to dentist, type of service, toothache, dental plaque, gingival bleeding, oral self-perception.
			Household income High > USD 450 Low ≤ USD 450	IRR (95% CI) 1.00 (ref) 1.57 (1.35–1.82)		
			Mother’s education High: > 8 years Low: ≤ 8 years	IRR (95% CI) 1.00 (ref) 1.19 (1.03–1.38)		
13	Page LA & Thomson WM [39] 2011 New Zealand Very high HDI 2003 – 0.908	Yes *Examiners* Baseline: 1 Follow-up: 1 (same)			ΔDF**S**	Sex, ethnicity.
			Composite SES (based on parents’ occupations): High Medium Low		Mean 0.0 (1.7) 0.4 (2.5) 0.9 (2.7)	
			Composite SES (area-based) High Medium Low		Mean 0.6 (3.5) 0.7 (2.3) 0.0 (2.6)	
14	Peres MA et al. [40]. 2018 Brazil Medium HDI 1999 – 0.663	Yes *Examiners* Baseline: 3 Follow-up: 1			Δ DM**T** > 0	Age, sex, birth weight, dmft at 6 years.
			Family income Stable low Downward Upward Stable high		IRR (95% CI) 2.28 (1.46–3.56) 1.78 (0.95–3.34) 1.62 (0.95–2.76) 1.00 (ref)	
					Δ F**T** > 0	
			Family income Stable low Downward Upward Stable high		IRR (95% CI) 0.55 (0.36–0.84) 1.05 (0.56–1.97) 0.75 (0.44–1.28) 1.00 (ref)	
					DMF**T** > 0	
			Family income Stable low Downward Upward Stable high		IRR (95% CI) 1.17 (0.83–1.65) 1.26 (0.76–2.08) 1.07 (0.70–1.62) 1.00 (ref)	
15	Pilecco RO et al. [41] 2020 Brazil High HDI 2012 – 0.732 High HD	Yes *Examiners* Baseline: 4 Follow-up: 4 (same)			ΔF**T** > 0	Sex, skin color, age, dentist visit, reason for visit, untreated dental caries.
			Father’s education High: > 8 year Low: ≤ 8 year		RR (95% CI) 1.00 (ref) 1.04 (0.96–1.12)	
			Household income High: ≥ US 450 Low: < US 450		RR (95% CI) 1.00 (ref) 0.79 (0.58–1.08)	
16	Saethre-Sundli HB et al. [42] 2020 Norway Very high HDI 2007 – 0.936	Yes *Examiners* Baseline: 44 Follow-up: 91 (some could be the same)		DMF**T** > 0		Gender, family status at 5 years of age, caries status at 5 years.
			Parents’ education High: Both high (> 12 years) Low: One or both low (≤ 12 yrs)	OR (95% CI) 1.00 (ref) 1.2 (1.0–1.4)		
			Parental background: Both Western One or both non-Western	OR (95% CI) 1.00 (ref) 1.2 (1.0–1.5)		
17	Schmoeckel J et al. [43] 2015 Germany Very high HDI 1995 – 0.858	Yes *Examiners* Baseline: 2 Follow-up: 1		ΔDMF**S** Low: DMF**S** ≤ .5 High: DMF**S**> 5		
			Father’s education: University degree Other	OR (95% CI) 1.00 (ref) 4.75 (CI not reported)		
			Mother’s education University degree Other	OR (95% CI) 1.00 (ref) 1.12 (CI not reported)		
			Type of school (participant): Gymnasium, 12 years’ schooling Other type of education	OR (95% CI) 1.00 (ref) 2.69 (CI not reported)		
18	Tagliaferro EP et al. [44] 2006 Brazil Medium HDI 1997 – 0.645	Yes *Examiners* Baseline: 2 Follow-up: 2 (same)		ΔDMF**S** > 0		dmfs, DMFS.
			Mother’s education: High > 8 years Low: ≤ 8 years	OR (95% CI) 1.00 (ref) 1.88 (1.03–3.45)		
19	Tan SHX et al. [45] 2021 Singapore Very high HDI 2009 – 0.910	NR *Examiners* Baseline: NR Follow-up: NR		ΔDMF**T** > 0		Gender, race, residence type, primary school region, baseline dmft and DMFT, baseline oral hygiene, tooth hypoplasia.
			Primary school type: Governmental Special Assistance Plan Others (including special education and home school)	RR (95% CI) 1.00 (ref) 1.03 (0.97–1.09) 1.94 (1.38–2.73)		
			Secondary school type Governmental Autonomous Independent Others (univariate analysis)	OR (95% CI) 1.00 (ref) 0.91 (0.86–0.97) 0.79 (0.72–0.87) 1.16 (0.95–1.42)		
20	Wang K et al. [46] 2020 China High HDI 2018 – 0.758	Yes *Examiners* Baseline: 3 Follow-up: 3 (same)	Calculated (Table 2)	ΔDMF**T**		
			Education (caregiver) High: ≥ 9 years Low: < 9 years	RR (95%) (ref) 0.85 (0.69–1.04)		
			Household monthly income: Low: < CNY 3000 Middle: CNY 3000–7000 High: > CNY 7000	RR (95%) 1.06 (0.82–1.36) 0.98 (0.78–1.23) 1.00 (ref)		
21	Warren JJ et al. [47] 2017 US Very high HD 1992 – 0.880/1995 – 0.886	Yes *Examiners* Baseline: NR Follow-up: NR		ΔDF**S** into 3 clusters:		Sex, juice intake, toothbrushing.
			Mother’s education Cluster 1: No incidence (4-year degree or more vs < 4-year degree) Cluster 2: Low incidence (4-year degree or more vs < 4-year degree) Cluster 3: High incidence (4-year degree or more vs < 4-year degree)	OR (95%) 1.00 (ref) 1.50 (0.91, 2.47) 2.05 (1.16, 3.60)		

SEK: Swedish kronor; USD: United States Dollar; CNY: Chinese yuan; MIH: Molar Incisor Hypomineralization; IRR: incidence rate ratio; OR: odds ratio; CI: confidence interval.

## Results of the syntheses

### Risk of bias in studies

The average quality score of the included studies was 6.98 points on this 10-point modified NOS for cohort studies (Table 2). The quality of individual studies ranged from medium to high, with scores of 4.50–9.75. Issues that reduced the quality of some studies included high attrition, which negatively affected representativeness, a too-short follow-up period, a lack of documentation of calibration before data collection, or missing results from caries reliability tests.

**Table 2:** The results from the adjusted Newcastle-Ottawa assessment form for cohort studies.

Study ID	Author (study), year	A Selection				B Comparability	C Outcome			Total score out of 10 possible
		Represent-ativeness of the exposed cohort	Selection of a non-exposed cohort	Ascertainment of exposure	Demonstration of outcome not present at start	0.25 points for each confounder adjusted for	Outcome assessment	Long enough follow-up (≥ 4 years) when enamel caries is not included (no period limit for enamel caries)	Adequacy of follow-up ≤ 20% lost)	
1	Alm A et al. [28], 2012	1	1	1	1	0.75	2	1	1	8.75
2	Bernabé E et al. [29], 2011	1	1	1	1	1.25	1	1	1	8.25
3	Brondani B et al. [30], 2024	0	1	1	1	1.50	1	1	0	6.50
4	Costa LR et al. [31], 2023	0	1	1	1	1.25	0.50	0	0	4.75
5	Gerdin EW et al. [32], 2008	1	1	1	1	0.50	1	1	1	7.50
6	Hall-Schullin E et al. [33], 2017	1	1	1	1	1.00	1	1	1	8.00
7	Julihn A et al. [34], 2010	1	1	1	1	2	1	1	1	9.00
8	Jung SH et al. [3], 2018	1	1	1	1	1.75	1	0	1	7.75
9	Källestål C et al. [35], 2007	1	1	1	1	1.75	2	1	1	9.75
10	Lena C et al. [36], 2020	1	1	1	1	1.25	0	1	1	7.25
11	Lu HX et al. [37], 2011	0	1	1	1	0.25	0.50	1	0	4.75
12	Ortiz FR et al. [38], 2022	0	1	1	1	1.75	1	1	0	6.75
13	Page, LA & Thomson WM. [39], 2011	0	1	1	1	0.50	1	0	0	4.50
14	Peres MA et al. [40], 2017	1	1	0	1	1	1	1	1	7.00
15	Pilecco RO et al. [41], 2020	0	1	1	1	1.50	0.50	0	0	5.00
16	Sæthre-Sundli HB et al. [42], 2020	1	1	1	1	0.75	2	1	1	8.75
17	Schmoeckel J et al. [43], 2015	0	1	1	1	0	1	1	0	5.00
18	Tagliafero EP et al. [44], 2006	0	1	1	1	0.50	0	1	0	5.50
19	Tan SHX et al. [45], 2021	1	1	1	1	2	0	1	1	8.00
20	Wang K et al. [46], 2020	1	1	1	1	0	1	0.5	1	6.50
21	Warren JJ et al. [47], 2017	1	1	1	1	0.75	0.50	1	1	7.25

### Summary of the evidence from the individual cohort studies

In Table 3, the various SES indicators are displayed: parental SES indicators (including composite SES indicators); adolescent-specific SES indicators (adolescents’ school type, social capital [proxy SES]); and immigrant background (proxy SES). Point estimates of OR, IRR, and RR and β coefficients, along with 95% CIs and SEs, most often quantify the association between SES and caries outcomes. Further details about the various SES composite indicators are presented in Appendix 1.7.

**Table 3:** Summary of evidence: impact of different SES indicators on dental caries in permanent teeth during the period 12–19 years.

ID	Type of SES indicator The Human Development Index (HDI):	Outcome Enamel caries: Included Excluded	Results
**Effect of parental SES indicators**			
**Parental education**			
*Mother’s education*			
7	Julihn et al. [34] Very high HDI	Excluded	When having a highly educated mother, individuals were less likely to experience proximal (contact between adjacent teeth) caries increment than those who had a mother with low education (adjusted OR: 0.73, 95% CI: 0.65–0.81).
21	Warren et al. [47] Very high HDI	Excluded	In the study, the individuals were categorized in clusters. Cluster 1 included participants with zero DFS at age 17 and no incidence from age 9 to 13 examinations. Cluster 3 included those with high DFS levels at 13 years and high incidence from 13 to 17 years. Participants in Cluster 3, compared to Cluster 1, had a higher OR of having mothers without a 4-year college degree. Cluster 3 was shown to be most consistently associated with lower maternal education (adjusted OR: 2.05, 95% CI: 1.16–3.60).
10	Llena et al. [36] Very high HDI	Included	Higher maternal education was associated with a lower DMFT than lower maternal education (adjusted OR: 0.37, 95% CI: 0.13–0.60).
		Excluded	Higher maternal education was associated with a lower DMFT than lower maternal education. (adjusted OR: 0.21, –0.13 – 0.54)
12	Ortiz et al. [38] High HDI	Excluded	Low maternal education was a risk factor for untreated caries (ΔDT) (adjusted IRR: 1.19, 95% CI: 1.03–1.38).
18	Tagliaferro et al. [44] Medium HDI	Excluded	Low maternal education was a predictor of caries increment in permanent dentition (ΔDMFS > 0) (OR: 1.88, 95% CI: 1.03–3.45).
17	Schmoeckel et al. [43] Very high HDI	Excluded	Caries increment (>Δ5 DMFS) in individuals was associated with low maternal education (OR: 1.12, 95% CI: not reported).
8	Jung et al. [3] Very high HDI	Excluded	No significant inverse relationship between high maternal educational level and untreated caries (DT > 0) was observed (adjusted OR: 0.86, 95% CI: 0.43–1.73).
*Father’s education*			
17	Schmoeckel et al. [43] Very high HDI	Excluded	Caries increment (>Δ5 DMFS) was associated with a father with low education (OR: 4.57, 95% CI not reported).
7	Juhlin et al. [34] Very high HDI	Excluded	Individuals with a highly educated father were less likely to experience proximal caries increment compared to those whose fathers had a low educational background (adjusted OR: 0.85, 95% CI: 0.77–0.95).
8	Jung et al. [3] Very high HDI	Excluded	Having a less educated father was considered a predictor of untreated caries (ΔDT) (adjusted OR: 2.04, 95% CI:1.05–3.98).
15	Pilecco et al. [41] High HDI	Excluded	No significant relationship was observed between fathers’ education and the increase in the number of filled teeth (ΔFT) (adjusted OR: 1.04, 95% CI: 0.96–1.12).
Education of both parents or caregiver (not reported if mother/father/other people*)			
16	Sæthre-Sundli HB et al. [42] Very high HDI	Excluded	Individuals with one or both parents having low education experienced a higher caries risk than those with both parents having a high level of education (OR: 1.2, 95% CI:1.0–1.4).
11	Lu et al. [37] Medium HDI	Excluded	No significant association between parental education level and DMFT in individuals was found (β coefficient: not reported, *p* > 0.05).
20	Wang et al. [46]High HDI	Excluded	No significant association between education level and the DMFT was found (RR: 0.85, 95% CI:0.69–1.04).
**Parental income** (household income is included)			
7	Julihn et al. [34] Very high HDI	Excluded	Low parental income showed a higher OR for having proximal caries increment than low parental income (adjusted OR: 1.15, 95% CI: 1.05–1.25).
12	Ortiz et al. [38] High HDI	Excluded	Low household income was a risk factor for untreated caries (ΔDT) (adjusted OR: 1.57. 95% CI: 1.35–1.82).
14	Peres et al. [40] Medium HDI	Excluded	An association was observed between caries and family income trajectories. The group of stable low-income showed a higher IRR of DMT compared to the group of stable high income (adjusted IRR: 2.28, 95% CI, 1.46–3.56) (referred to the article’s Table 2)
11	Lu et al. [37] Medium HDI	Excluded	No significant association between the level of monthly household income and DMFT was found (β coefficient: not reported, *p* > 0.05).
15	Pilecco et al. [41] High HDI	Excluded	No significant association between low household income and increase in the number of filled teeth (ΔFT) (adjusted RR: 0.79, 95% CI: 0.58–1.08)
20	Wang et al. [46] High HDI	Excluded	No significant association between low household income and caries (ΔDMFT) was observed (adjusted RR: 1.06, 95% CI: 0.82–1.36)
**Parental occupation**			
9	Källestål et al. [35] Very high HDI	Included	The risk of caries increment (ΔDMFS) was higher among individuals from working-class households than among those from civil servant households (adjusted RR: 1.06, 95% CI: 1.01–1.12)
		Excluded	The risk of caries increment (ΔDMFS) was higher among individuals from working-class households than among those from civil servant households (adjusted RR: 1.04, 95% CI: 1.02–1.07).
**Composite SES indicators**			
3	Brondani et al. [30] High HDI	Excluded	Low individual SES was directly associated with a higher caries experience (DMT) at follow-up (adjusted β: –0.28, SE: 0.10).
4	Costa et al. [31] High HDI	Excluded	Lower SES indirectly predicted caries incidence after adjustment via sugar consumption and inactive lifestyle (adjusted β: –0.046, SE: 0.023).
13	Page et al. [39] in Very high HDI	Excluded	The high-deprivation group had a high ΔDFS (ΔDFS: 0.6, SD:3.5), while the low-deprivation group had a low ΔDFS (ΔDFS: 0.0, SD:2.6). Participants with low household SES had a high ΔDFS (ΔDFS: 0.9, SD:2.7) compared with high household SES (ΔDFS: 0.0, SD:1.7).
6	Hall-Schullin et al. [33] Very high HDI	Excluded	SES was associated with higher DMFT in the most deprived area compared to the least deprived area used as the reference (adjusted IRR: 1.77, 95% CI: 1.41–2.23).
5	Gerdin et al. [32] Very High HDI	Excluded	The individuals were classified into 5 distinct socioeconomic clusters ranging from low (cluster V) to high (cluster I). At age 12, DFT was almost twice as high in the lowest SES cluster compared to the highest one (cluster V (low): DFT: 1.1, cluster I (high): DFT: 0.6, *p* < 0.002).
8	Jung et al. [3] Very high HDI	Excluded	Low SES was not significantly associated with untreated caries (ΔDT) (adjusted OR: 1.80, 95% CI: 0.88–3.64).
**Effect of adolescent-specific SES indicators**			
*Adolescents’ school type*			
17	Schmoeckel et al. [43] Very high HDI	Excluded	School type was defined as either ‘gymnasium’, a scholar pathway after elementary school of 4–6 years preparing for future university studies, or ‘other type of education’. In the last category, individuals were more likely to belong to the group with high caries increment (DMFS ≥ 5) (OR: 2.69, 95% CI: not reported).
8	Jung et al. [3] Very high HDI	Excluded	Individuals with vocational education were more likely to experience untreated caries (ΔDT) compared with those with general education, for whom college education was possible afterwards (OR: 2.68, 95% CI: 1.46–4.92).
19	Tan et al. [45] Very high HDI	Excluded	Three groups of primary school types: Governmental Special Assistance Plan (SAP) schools (with bilingual and bicultural students), non-SAP governmental schools, and ‘others’ (including madrasah schools, special education schools, and home schooling). Enrollment in the ‘others’ category was a predictor of caries incidence (adjusted RR: 1.94, 95% CI: 1.38–2.73).
*Adolescents’ social capital (proxy SES)*			
2	Bernabé et al. [29] Very high HDI	Excluded	‘Support from a special person’ (‘Special person factor score’) was associated with a lower DMFT in second permanent molars. The DMFT value decreased by a factor of 0.81 per SD increase in this score. (adjusted RR: 0.81, 95% CI: 0.69–0.96).
4	Costa et al. [31] High HDI	Excluded	Lower social support indirectly predicted caries incidence via sugar consumption and physical inactivity (adjusted β: –0.026, Bootstrap SE: 0.015).
**Effect of immigrant background (proxy SES)**			
9	Källestål et al. [35] Very high HDI	Included	Individuals born in Eastern Europe (lower HDI than Sweden) had an elevated risk for caries increment compared with adolescents born in Sweden (adjusted RR: 1.25, 95% CI: 1.03–1.50)
		Excluded	Individuals born in Eastern Europe (lower HDI than Sweden) had an elevated risk for caries increment compared with adolescents born in Sweden (adjusted RR: 1.25, 95% CI: 1.12–1.40)
7	Julihn et al. [34] Very high HDI	Excluded	Individuals born in Eastern Europe (lower HDI than Sweden) had an elevated caries risk for proximal caries increment compared with individuals born in Sweden (OR: 1.44, 95% CI: 1.12–1.85).
1	Alm et al. [28] Very high HDI	Included	The findings of caries on proximal surfaces indicated that individuals with both parents ‘born abroad’ were more likely to experience high caries increment (DFproxS ≥ 4) compared to those with ‘both parents born in Sweden’ (adjusted OR 3.7, 95% CI: 1.1–12.8).
16	Sæthre-Sundli et al. [42] Very high HDI	Excluded	Individuals living with one or both parents from non-Western countries had higher odds ratios of having DMFT compared to those living with both mother and father of Western origin (adjusted OR: 1.2, 95% CI: 1.0–1.4).

DFS: decayed filled surfaces; DMFT: Decayed, Missing, and Filled Tooth; DT: decayed teeth; FT: filled teeth; SES: socioeconomic status; RR: relative risk; OR: odds ratio; CI: confidence interval; IRR: incidence rate ratio; SD: standard deviation.

## Discussion

This systematic review synthesized evidence from 21 cohort studies across 11 countries to evaluate the association between SES and dental caries in adolescents. By restricting inclusion to cohort designs and to adolescents aged 12–19 years, the review provides age-specific insights into socioeconomic gradients in caries without dilution from childhood or adult data. Across most analyses, SES indicators were associated with caries outcomes, supporting the relevance of socioeconomic conditions for oral health during adolescence.

### Interpretation of main findings

Among the SES indicators assessed, maternal education showed the most consistent association with caries outcomes, with six of seven studies reporting an inverse relationship [34, 36, 38, 43, 44, 47]. Associations with fathers’ education and parental income were observed less consistently, with an inverse association of fathers’ education reported in three out of four studies [3, 34, 43] and of parental income in three out of six studies [34, 38, 40]. These findings contrast with earlier research suggesting that traditional parental SES indicators are weakly or inconsistently related to adolescent health outcomes [18, 19], but align with evidence indicating that parental SES indicators influence caries into adolescence.

Composite SES measures consistently demonstrated socioeconomic gradients in caries across five out of six included studies [30–33, 39], supporting the notion that multidimensional indicators may better capture socioeconomic influences than single variables. SES indicators linked directly to adolescents – such as school type [3, 43, 45] and social capital [29, 31] – also showed meaningful associations, albeit in fewer studies. These findings are particularly relevant given adolescents’ increasing independence and the growing influence of peer, school, and neighborhood contexts on health behaviors [11, 12].

### Methodological heterogeneity and certainty of evidence

The initial stage of the screening process, phase 1 (title/abstract screening), followed best-practice guidance of full dual-review only in 10% of the publications. Although one reviewer screened the publications three times, and all publications classified as ‘*maybe*’ in phase 2 (further screening process) were read and assessed by both reviewers, this constituted a methodological limitation and a risk that some publications could have been overlooked.

A major challenge in interpreting the evidence was the substantial methodological heterogeneity across studies. Differences in caries outcome definitions (incidence versus increment, variation in the use of DMFT or DMFS index, inclusion or exclusion of enamel lesions), SES operationalization, and effect measures (OR, RR, IRR, β coefficients) limited comparability and precluded meta-analysis. Although exclusion of the ‘M’ component likely had minimal impact because extractions due to caries are generally rare among adolescents [48], inconsistent handling of enamel caries, which accounts for a large proportion of adolescent caries experience [14], may have biased estimates toward underestimation. Concerning a lacking ‘F’ component, it may have introduced bias mostly restricted to data in late adolescence, as the median time for caries progression from the onset of caries into the dentin layer in permanent teeth is 4 years [26]. This component is also ambivalent. In countries without free dental health care, restorative treatment might be limited to those whose parents can afford it.

Heterogeneity was further compounded by variation in follow-up duration, examiner calibration, and use of BW radiographs. These methodological inconsistencies, together with relatively small sample sizes in many studies (only two individual studies had a baseline sample size of more than 10,000), reduced the precision of effect estimates and the overall certainty of evidence.

Residual confounding is also likely to have influenced observed associations. The confounders most often adjusted for were sex, ethnicity, and baseline caries. Contextual factors such as water fluoridation, access to dental care, and broader oral healthcare systems were less frequently considered.

The use of a modified NOS for quality assessment made it impossible to compare our scores with other systematic reviews using the standard NOS. Therefore, the scores of two identified systematic reviews targeting adults, using the standard NOS [15, 49], could not be compared with our review, although their scores were approximate.

### Equity, generalizability, and external validity

The generalizability of the findings is limited by the geographical and socioeconomic distribution of included studies. Most were conducted in very high-HDI countries, and none of the studies was conducted in countries with a low HDI. Given documented differences in healthcare systems, preventive measures (e.g. fluoridation), and social determinants of health, the SES–caries associations observed here may not directly apply to lower-income contexts.

Additionally, the exclusion of socioeconomically deprived groups, including Indigenous and tribal populations, restricts external validity and may underestimate the true magnitude of socioeconomic inequalities in adolescent caries. Similarly, the exclusion of grey literature [50] may have contributed to publication bias. While these decisions enhanced methodological comparability, they simultaneously highlighted persistent inequities in the available evidence. Inclusive research strategies should therefore be promoted.

### Potential mechanisms through which SES indicators affect caries in adolescents

It is acknowledged that a broad range of underlying determinants interact when assessing for association between SES and caries, for example, biological, behavioral, psychosocial, and economic [9].

#### Behavioral pathways

Compared to having a low educational background, individuals with a higher educational background are more easily able to make healthier diet choices and understand the importance of oral hygiene and preventive oral care, which means a higher level of oral health literacy. Adolescents’ diet is higher in sugars compared with the diet of children and adults [51], has a pattern of frequent snacking between meals [52], and adolescents’ oral hygiene is occasionally poorer than that of the other age groups [53]. SES indicators targeting these challenges in adolescents by using behavioral pathways should be widely adopted. Adolescents living in low socioeconomic environments also have an elevated risk of additional unhealthy behaviors, such as early initiation of smoking and involvement in drug abuse [54]. The mechanisms through which SES indicators affect caries in adults and children may differ due to other aspects of everyday life, but also, for example, in the case of adults, the enamel of permanent teeth is now fully matured and not to the same extent prone to caries. Regarding future intervention for adolescents, oral health education must be adjusted and based on credibility and confidence [55].

#### Access pathways

Across the world, not all adolescents live in societies with access to preventive and restorative dental care, and if such access exists, not all can afford services, such as access to oral health literacy skills and fluoridation. However, in some areas, water fluoridation is available, reducing the caries risk.

#### Psychosocial pathways

Adolescents, characterized by being in psychological and social transitions, are vulnerable to different psychosocial challenges, including depression [11], and are more often affected than children and adults. SES proxies for caries, such as social capital or social support, may help to uncover those who struggle, which is important as poor oral health is associated with low SES [56]. Living in countries without a functioning oral healthcare system may be detrimental to the oral health of those adolescents.

Considering our study, unmeasured behavioral and psychosocial factors, such as oral health literacy or oral health-seeking behavior, may have resulted in both over- and underestimation of SES–caries associations, reinforcing the need for cautious interpretation and non-causal language.

### Implications for research and practice

Adolescence is a critical period for caries initiation, particularly in the context of enamel lesions [57]. Therefore, when enamel caries is included in the caries diagnosis, the conclusions drawn by researchers that new caries occurs at a relatively constant rate and that adolescence is not a period of heightened caries risk [58] may not hold. Evidence suggests that SES indicators are most informative during this stage, when newly erupted permanent teeth are highly susceptible [59]. The consistent performance of composite SES indicators in predicting caries supports calls for developing standardized, adolescent-specific SES measures, as single indicators may insufficiently capture complex social exposures [60].

Existing composite SES scales for children [61] and adults [62] provide a foundation, but importantly, adolescent-specific scales should incorporate both parental factors and adolescent-contextual indicators, including school environment, social capital, and peer influences. Or, in other words, such scales should reflect how adolescents live their lives in different regions of the world today. For adolescents living in socioeconomically disadvantaged and vulnerable populations, some scale adjustments may be considered, for example, to clarify whether they have the financial means to pay for dental treatment and oral hygiene cleaning products, such as toothbrushes and fluoride toothpaste. Future research should prioritize prospective cohort designs, include disadvantaged socioeconomic populations, and apply multilevel analytical approaches to disentangle individual and contextual effects [15]. To address the multidimensional, latent, and context-dependent nature of SES, no single method is sufficient on its own.

## Conclusions

Our findings revealed considerable heterogeneity across the included studies, which precluded meta-analysis and limited the strength of definitive conclusions. As a result, the overall certainty of evidence regarding the association between SES and caries in adolescents remains low. Nevertheless, the results consistently suggested an inverse association between maternal education and caries, while inverse associations with fathers’ education and parental income were weaker and less consistent. This type of association was also observed for adolescents’ school type, their social capital, and composite SES indicators. Immigrant background served as a caries predictor. The findings underscore the need to develop and validate robust composite SES measures applicable across diverse global contexts.

## Supplementary Material



## Data Availability

All data generated and analyzed in this study are included in this published article and its Supporting Information file.
