# Supplementary material for: Association between socioeconomic status and dental caries in adolescents – a systematic review of cohort studies
Source: Acta Odontol Scand. 2026 Oct 7;85:46953. doi: 10.2340/aos.v85.46953 (PMC13651455; doi:10.2340/aos.v85.46953)
Supplement: Supplementary file 1 [file AOS-85-46953-s1.pdf]

Supplementary material has been published as submitted. It has not been copyedited or typeset by Acta Odontologica Scandinavica.

Appendix

	Contents
Word document, 533 KB	**Appendix 1. 1 a.** Meta-analysis. Effect of mother's education on caries incidence among adolescents.
	**Appendix 1. 1 b.** Funnel plot to assess publication bias – Effect of Mother's education.
	**Appendix 1. 1 c.** Meta-analysis. Effect of parental income on caries incidence among adolescents.
	**Appendix 1. 2.** Exclusion criteria for systematic review.<br>**Appendix 1.3.** Search strategy in four electronic databases: Medline, Embase, Cochrane Library, and Web of Science.<br>**Appendix 1. 4.** Modified Newcastle-Ottawa Quality Assessment Form for Cohort Studies.<br>**Appendix 1. 5** List of excluded publications and the main reason(s) for exclusion (n = 46).<br>**Appendix 1. 6.** List of publications covering the same cohort studies and how the included publications were selected. References for Appendix 1.5 and Appendix 1.6 are included.<br>**Appendix 1. 7.** Definitions and integration methods of composite SES<br>.

Appendix 1. 1 a.

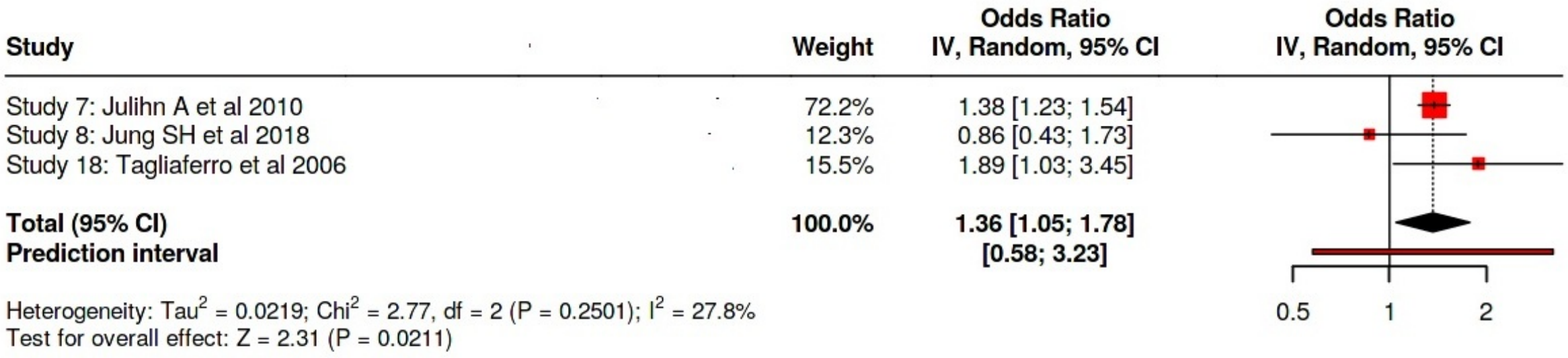

Meta-analysis. Effect of mother's education on caries incidence among adolescents.

Appendix 1. 1 b

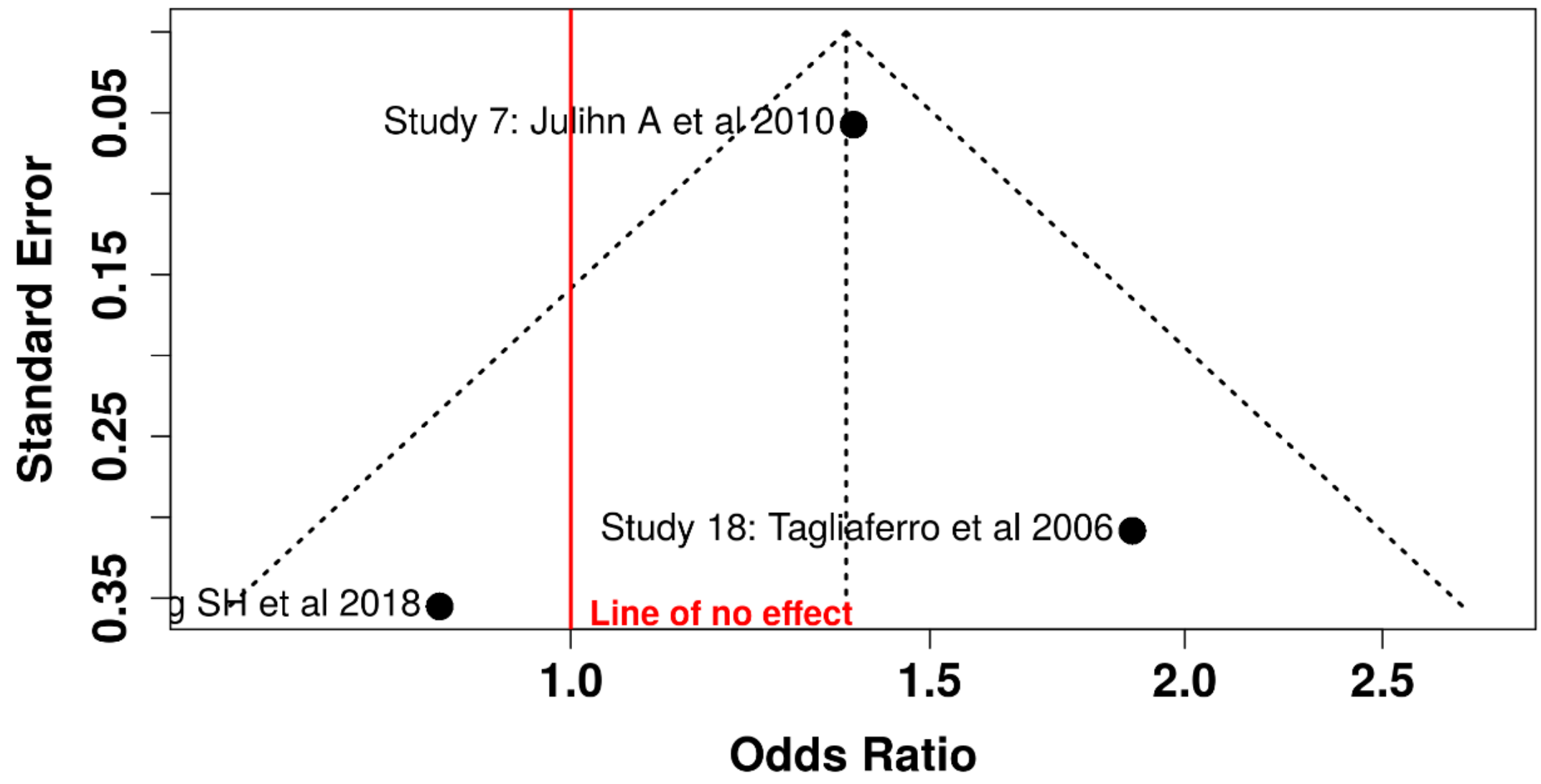

Funnel plot to assess Publication Bias – Effect of Mother's education.

Appendix 1. 1 c.

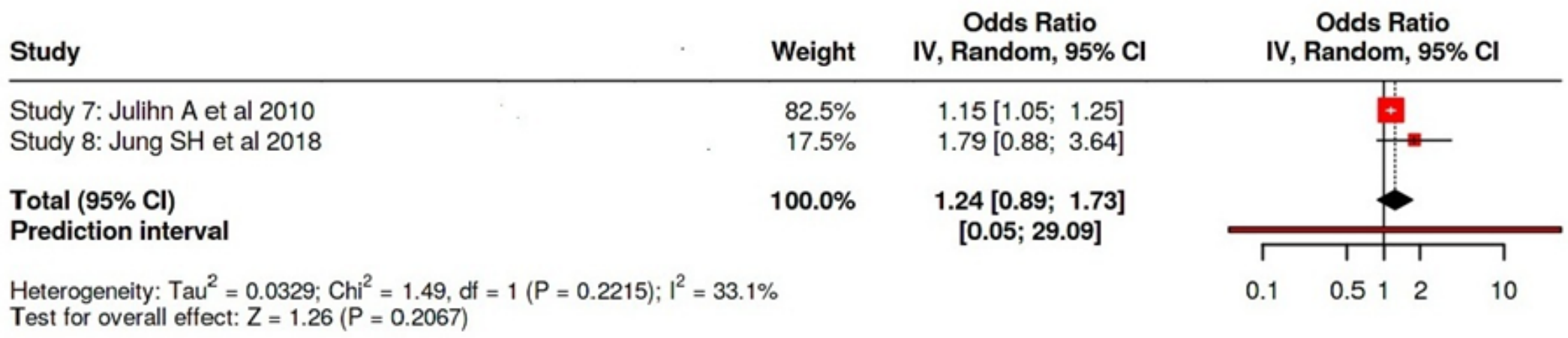

Meta-analysis. Effects of parental income on caries incidence among adolescents.

Appendix 1. 2. Exclusion criteria for systematic review.

**Exclusion criteria for the systematic review**
Other languages than English and Scandinavian languages<br>The final caries assessment for the calculation of the association was not performed during ages 12- 19<br><br>Studies published before 2000 and after December 5th, 2025.<br><br>Reviews.<br><br>Meta-analyses.<br><br>Theses.<br><br>Ecological studies.<br><br>Grey literature.<br><br>Not representative populations, such as socioeconomically deprived groups (Indigenous or tribal groups), dental students, or groups with various medical problems.<br><br>Studies solely used neighborhood or geographic background without any further SES measurements.<br><br>Intervention studies that might influence the association between SES and caries.<br><br>Studies using SES estimates based on self-estimation (*e.g.* satisfaction with their SES or what they believed their SES constituted), self-reported measurements about caries status or oral-health-related quality of life (OHRQoL), as well as assessed measurements about OHRQoL.<br><br>When different publications reported the same study, only one publication was selected. The others were excluded.

Appendix 1. 3. Search strategy in four electronic databases: Medline, Embase, Cochrane Library, and Web of Science.

Ovid MEDLINE(R) and Epub Ahead of Print and In-Process, In-Data-Review & Other Non-Indexed Citations and Daily <1946 to December 05, 2025>

Date: 08.12.2025

1 exp Dental Caries/ 53693

2 (Caries or carious or ((dentin* or dental or tooth or teeth) adj3 (lesion* or cavit* or decay* or white spot*))).ti,ab,kf. 68446

3 1 or 2 81999

4 exp Socioeconomic Factors/ or exp Health Inequities/ or "Social Determinants of Health"/ or exp Social Class/ or Income/ or exp Poverty/ or Educational Status/ or exp "Emigrants and Immigrants"/ 594360

5 (SES or SEP or socioeconomic* or socio-economic* or socio economic* or social economic* or social inequalit* or social inequit* or health inequit* or health inequalit* or social factor* or social class or social capital or social determinant* or educational status or educational level* or educational attainment* or educational achievement* or economic status or economic factor* or poverty or income or wealth* or occupation* or employ* or unemploy* or ethnic* or immigrant* or emigrant* or migrant* or neighbourhood or neighborhood or home owner* or homeowner*).ti,ab,kf. 1936668

6 4 or 5 2241369

7 exp Cohort Studies/ or exp Randomized Controlled Trial/ 3319582

8 (cohort or incidence stud* or concurrent stud* or follow up stud* or follow-up stud* or longitudinal stud* or longitudinal survey* or prospective stud* or retrospective stud* or RCT or randomized or randomised or randomly or placebo).ti,ab,kf. 2931811

9 trial.ti. 353787

10 (randomized controlled trial or controlled clinical trial).pt. 743351

11 7 or 8 or 9 or 10 4812052

12 young adult/ or Adolescent/ 2927898

13 (adolescen* or teen* or youth or young adult* or puber* or pubescen* or 12 year* or 13 year* or 14 year* or 15 year* or 16 year* or 17 year* or 18 year* or 19 year* or 12-year* or 13-year* or 14-year* or 15-year* or 16-year* or 17-year* or 18-year* or 19-year* or school or student* or pupil* or school-age or schoolage or permanent tooth or permanent teeth or permanent dentition).ti,ab,kf. 1762203

14 12 or 13 3926716

15 3 and 6 and 11 and 14 1010

16 exp animals/ not humans/ 5403064

17 limit 15 to (case reports or meta analysis or "review" or "systematic review") 52

18 15 not (16 or 17) 957

19 limit 18 to yr="2000 -Current" 850

https://ovidsp.ovid.com/ovidweb.cgi?T=JS&NEWS=N&PAGE=main&SHAREDSEARCHID=3m3Mw23WIMbwPkjFxOuLK4NnyUFZVN9c1GDA8x2CGUGkYqqJziKNyLA7SegOuG7Fy

Embase <1974 to 2025 December 04>

Date: 08.12.2025

1 dental caries/ 64392

2 (Caries or carious or ((dentin* or dental or tooth or teeth) adj3 (lesion* or cavit* or decay* or white spot*))).ti,ab,kf. 67323

3 1 or 2 84372

4 exp "social aspects and related phenomena"/ or "social determinants of health"/ or exp socioeconomics/ or exp social status/ or occupation/ or exp employment/ or exp income/ or exp migrant/ 4796736

5 (SES or SEP or socioeconomic* or socio-economic* or socio economic* or social economic* or social inequalit* or social inequit* or health inequit* or health inequalit* or social factor* or social class or social capital or social determinant* or educational status or educational level* or educational attainment* or educational achievement* or economic status or economic factor* or poverty or income or wealth* or occupation* or employ* or unemploy* or ethnic* or immigrant* or emigrant* or migrant* or neighbourhood or neighborhood or home owner* or homeowner*).ti,ab,kf. 2408708

6 4 or 5 6314842

7 exp randomized controlled trial/ or cohort analysis/ 2580655

8 (cohort or incidence stud* or concurrent stud* or follow up stud* or follow-up stud* or longitudinal stud* or longitudinal survey* or prospective stud* or retrospective stud* or RCT or randomized or randomised or randomly or placebo).ti,ab,kf. 4692005

9 trial.ti. 574198

10 7 or 8 or 9 5291164

11 exp adolescent/ or young adult/ 2472832

12 (adolescen* or teen* or youth or young adult* or puber* or pubescen* or 12 year* or 13 year* or 14 year* or 15 year* or 16 year* or 17 year* or 18 year* or 19 year* or 12-year* or 13-year* or 14-year* or 15-year* or 16-year* or 17-year* or 18-year* or 19-year* or school or student* or pupil* or school-age or schoolage or permanent tooth or permanent teeth or permanent dentition).ti,ab,kf. 2452808

13 11 or 12 3981411

14 3 and 6 and 10 and 13 1535

15 exp animal/ not exp human/ 5885531

16 limit 14 to (conference abstract or conference paper or "conference review" or "review")131

17 limit 14 to (meta analysis or "systematic review") 76

18 14 not (15 or 16 or 17) 1363

19 limit 18 to yr="2000 -Current" 1288

20 limit 19 to "remove clinical trial (clinicaltrials.gov) records" 1016

https://ovidsp.ovid.com/ovidweb.cgi?T=JS&NEWS=N&PAGE=main&SHAREDSEARCHID=39p7ighkQyvF0roSzYyP8abxGEBoTC2Njldg5Cj2D9J6gqHTtF1EpL8na8guwXGpD

Cochrane Library

Content: Cochrane Central Register of Controlled Trials, Issue 11 of 12, November 2025

Date: 08.12.25

ID Search Hits

#1 [mh "Dental Caries"] 3942

#2 (Caries OR carious OR ((dentin* OR dental OR tooth OR teeth) NEAR/3 (lesion* OR cavit* OR decay* OR white NEXT spot*))):ti,ab,kw 13494

#3 #1 OR #2 13494

#4 [mh "Socioeconomic Factors"] OR [mh "Health Inequities"] OR [mh ^"Social Determinants of Health"] OR [mh "Social Class"] OR [mh ^"Income"] OR [mh "Poverty"] OR [mh ^"Educational Status"] OR [mh "Emigrants and Immigrants"] 15406

#5 ("SES" OR "SEP" OR socioeconomic* OR socio NEXT economic* OR social NEXT economic* OR social NEXT inequalit* OR social NEXT inequit* OR health NEXT inequit* OR health NEXT inequalit* OR social NEXT factor* OR "social class" OR "social capital" OR social NEXT determinant* OR "educational status" OR educational NEXT level* OR educational NEXT attainment* OR educational NEXT achievement* OR "economic status" OR economic NEXT factor* OR "poverty" OR "income" OR wealth* OR occupation* OR employ* OR unemploy* OR ethnic* OR immigrant* OR emigrant* OR migrant* OR neighbourhood OR neighborhood OR home NEXT owner* OR homeowner*):ti,ab,kw 109246

#6 #4 OR #5 111511

#7 [mh ^"young adult"] OR [mh ^"Adolescent"] 193882

#8 (adolescen* OR teen* OR youth OR young adult* OR puber* OR pubescen* OR 12 NEXT year* OR 13 NEXT year* OR 14 NEXT year* OR 15 NEXT year* OR 16 NEXT year* OR 17 NEXT year* OR 18 NEXT year* OR 19 NEXT year* OR school OR student* OR pupil* OR school-age OR schoolage OR permanent tooth OR permanent teeth OR permanent dentition):ti,ab,kw 431222

#9 #7 OR #8 431222

#10 #3 AND #6 AND #9 with Cochrane Library publication date Between Jan 2000 and Dec 2025, in Trials 348

https://www.cochranelibrary.com/advanced-search/search-manager?search=7890810

Web of Science Core Collection

Date: 08.12.2025

Entitlements: WOS.SCI: 1945 to 2024, WOS.AHCI: 1975 to 2024, WOS.ESCI: 2019 to 2024, WOS.SSCI: 1956 to 2024

#1 Search: TS=("Caries" OR "carious" OR (("dentin*" OR "dental" OR "tooth" OR "teeth") NEAR/2 ("lesion*" OR "cavit*" OR "decay*" OR "white spot*")))

Results: 62006

#2 Search: TS=("SES" OR "SEP" OR "socioeconomic*" OR "socio-economic*" OR "socio economic*" OR "social economic*" OR "social inequalit*" OR "social inequit*" OR "health inequit*" OR "health inequalit*" OR "social factor*" OR "social class" OR "social capital" OR "social determinant*" OR "educational status" OR

"educational level*" OR "educational attainment*" OR "educational achievement*" OR "economic status" OR "economic factor*" OR "poverty" OR "income" OR "wealth*" OR "occupation*" OR "employ*" OR "unemploy*" OR "ethnic*" OR "immigrant*" OR "emigrant*" OR "migrant*" OR "neighbourhood" OR "neighborhood" OR "home owner*" OR "homeowner*")

Results: 4141351

#3 Search: TS=("cohort" OR "incidence stud*" OR "concurrent stud*" OR "follow up stud* OR "follow-up stud* OR "longitudinal stud*" OR "longitudinal survey*" OR "prospective stud*" OR "retrospective stud*" OR "RCT" OR "randomized" OR "randomised" OR "randomly" OR "placebo")

Results: 3383479

#4 Search: TI=("trial")

Results: 484631

#5 Search: #3 OR #4

Results: 3543558

#6 Search: TS=("adolescen*" OR "teen*" OR "youth" OR "young adult*" OR "puber*" OR "pubescen*" OR "12 year*" OR "13 year*" OR "14 year*" OR "15 year*" OR "16 year*" OR "17 year*" OR "18 year*" OR "19 year*" OR "12-year*" OR "13-year*" OR "14 -year*" OR "15-year*" OR "16-year*" OR "17-year*" OR "18-year*" OR "19-year*" OR "school" OR "student*" OR "pupil*" OR "school-age" OR "schoolage" OR "permanent tooth" OR "permanent teeth" OR "permanent dentition")

Results: 2677407

#7 Search: (#1 AND #2 AND #5 AND #6) NOT (DT==("PROCEEDINGS PAPER"))
Timespan: 2000-01-01 to 2026-01-01

Results: 603

https://www.webofscience.com/wos/woscc/summary/db477079-82c4-4e3b-b1b6-e3d95db87b06-018f59d0bb/relevance/1

Appendix 1. 4. The modified Newcastle-Ottawa Quality Assessment Form for Cohort Studies

Selection
1) Representativeness of the exposed cohort<br>a) Truly representative (one point)<br>b) Somewhat representative (one point)<br>c) Selected group<br>d) No description of the derivation of the cohort<br>2) Selection of the non-exposed cohort<br>a) Drawn from the same community as the exposed cohort (one point)<br>b) Drawn from a different source<br>c) No description of the derivation of the non-exposed cohort<br>3) Ascertainment of exposure<br>a) Standardised record/use of known measurements (one point)<br>b) Validated questionnaires/structured interviews (one point)<br>c) Written self-report<br>d) No description<br>e) Other<br>4) Demonstration that the outcome of interest was not present at the start of the study<br>a) Yes (one star)<br>b) No
Comparability
1) Comparability of cohorts based on the design or analysis controlled for confounders<br>a) The study controls: 0.25 points for each confounder (up to a total of 2 points).<br>b) Cohorts are not comparable based on the design or analysis controlled for confounders
Outcome
1) Assessment of outcome<br>a) Calibrated examiners (0.50 points) with reliability caries examination tests presented (0.50 points)<br>b) Use of radiographs (bitewings) (one point).<br>c) Self-report<br>d) No description<br>e) Other<br>2) Was the follow-up long enough for outcomes to occur?<br>a) Yes (one point)<br>b) No<br>Indicate the median duration of follow-up and a brief rationale for the assessment above: 10-11 years up to 17 years: 4 years. 17 years and more: 7 years. For enamel caries, no limit is set for the follow-up period.<br>3) Adequacy of follow-up of cohorts<br>a) Complete follow-up- all subjects accounted for (one point)<br>b) Subjects lost to follow-up unlikely to introduce bias- number lost less than or equal to 20% or description of those lost suggested no different from those followed. (one point)<br>c) Follow-up rate less than 80% and no description of those lost<br>d) No statement

Note: A study can be given a maximum of one point in the Selection category, while a maximum of two points for the categories Comparability and Outcome.

The changes from the original Newcastle-Ottawa Scale (NOS) were as follows: In the summary calculation, stars were changed to points. In Selection- part 3 “Ascertainment of the exposure”, the original text in a) and b) was modified to a) standardized record/use of known measurements (1 point), b) validated questionnaires/structured interviews (1 point). In the section “Comparability”, the text was altered to 0.25 points for each confounder (a maximum of 2 points could be reached). Likewise, as the text in Outcome, part 1) Assessment of the outcome a) and b), was not relevant for caries assessment, the text was changed to a) calibration of examiners (0.5 point), presented reliability caries tests (0.5 point), b) use of radiographs (bitewings) (1 point).

Appendix 1. 5. List of excluded publications and the main reason(s) for exclusion (n = 46).

First author Year	*The main reason(s) for exclusion*
Alves FN. [1] 2015	Not a cohort.
Birkeland JM. [2] 2002	Association SES – caries, not studied.
Brusius CD.[3] 2023	Association SES – caries, not studied.
Borowska-Struginska, B. [4] 2016	Not a cohort.
Broadbent JM. [5] 2016	Association SES – caries, not studied.
Brondani, B et al. [6] 2025	Insufficient SES or outcome variables.
Carbajal Rodriguez, G. [7] 2024	Not a cohort (only one caries examination).
Cianetti S. [8] 2018	Not a cohort (a systematic review).
Cianetti S. [9] 2017	Not a cohort (only one caries examination).
David J [10] 2006	Missing relevant data (no values about the association between SES and caries).
Ditmyer M. [11] 2008	Not a cohort.
Ditmyer, M.[12] 2011	Not a cohort.
Divaris K.[13] 2012	Final caries assessment not during 12-19 yrs.
Ghazal TS. [14] 2019	Missing relevant data (due to not having a representative group).
Goncalves BML et al. [15] 2025	Insufficient SES or outcome variables.
Haugejorden O. [16] 2006	Association SES – caries, not studied.
Holmen A. [17] 2013	Association SES – caries, not studied.
Holst D. [18] 2012	Final caries assessment not during 12-19 yrs.
Hummel R. [19] 2021	Final caries assessment not during 12-19 yrs. (inclusion of increment measurements before the age of 12 years).
Khoramrooz, M. [20] 2004	Final caries assessment not during 12-19 yrs. (The majority of the individuals did not have permanent teeth).
Lew LH et al. [21] 2024	Insufficient SES or outcome variables.
Lewsey JD. [22] 2004	Final caries assessment not during 12-19 yrs.
Lieske, B et al. [23] 2025	Not a cohort.
Masood M. [24] 2014	Insufficient SES or outcome variables.
Meira GF. [25] 2020	Association SES – caries, not studied.
Meyer K [26] 2010	Not a cohort (intervention study).
Nicolau B [27] 2005	Not a cohort (only one caries examination).
Nishioka, D.,[28] 2021	Insufficient SES or outcome variables.
Noro LR [29] 2015	Missing relevant data (the exact association between SES and caries incidence was not referred to).
Noronha-Ramos, TG et al. [30] 2025	Insufficient SES or outcome variables.
Ogwo, C. [31] 2023	Final caries assessment not during 12-19 yrs.
Östberg AL. [32]2020	Final caries assessment not during 12-19 yrs. (inclusion of increment measurements before the age of 12 years).
Peres KG.[33] 2009	Insufficient SES or outcome variables.
Peres MA.[34] 2007	Not a cohort (only one caries examination).
Peres KG. [35] 2011	Final caries assessment not during 12-19 yrs.
Ruiz B [36] 2023	Final caries assessment not during 12-19 yrs.
Schmidt J [37] 2022	Not a cohort.

Sousa FS [38] 2021	Not a cohort (only one caries examination).
Stangvaltaite L. [39] 2015	Insufficient SES or outcome variables.
Swedberg. Y, [40] 2003	Final caries assessment not during 12-19 yrs.
Taani, D. Q [41] 2002	Not a cohort.
Teixeira AK [42] 2016	Final caries assessment not during 12-19 yrs.
Teixeira AKM [43] 2019	Final caries assessment not during 12-19 yrs.
Thomson WM [44] 2000	Final caries assessment not during 12-19 yrs.
Virtanen, JI [45] 2001	Association SES – caries, not studied.
Ying Y et al. [46]2025	Insufficient SES or outcome variables.

Appendix 1. 6. List of publications covering the same cohort studies and how the included publications were selected.

Study	*Various publications*	*Included publication*	*Study ID*	*Reasons for selection*
Study from the Municipality of Jönköping	Alm A et al. 2012 [47]<br>Alm A et al. 2008 [48]	Alm A et al. 2012	1	The most recent publication.
Nationwide Swedish public dental health clinics	Källestål C et al. 2007 [49]<br>Källestål C et al. 2002 [50]	Källestål C et al. 2007	9	The most recent publication.
Substudy of the Pelotas study from 1993	Peres MA et al. 2018 [51]<br>Peres MA et al. 2009 [52]	Peres MA et al. 2018	14	The most recent publication.
School children from Porto Alegre, South Brazil	Ortiz FR et al. 2022 [53]<br><br>Ortiz FR et al. 2020 [54]	Ortiz FR et al. 2022	12	The most recent publication.
Children attended primary health care units on the National Children's Vaccination Day in the municipality of South Santa Maria, Brazil	Brondani B et al. 2024 [55]<br>Knorst JK et al. 2022 [56]	Brondani B et al. 2024	3	The most recent publication.
Birth cohort study from Iowa Fluoride Study (IFS)	Warren JJ et al. 2017 [57]<br>Curtis AM et al. 2018 [58]<br>Chankanka O et al. 2011 [59]<br>Broffitt B et al. 2013 [60]	Warren JJ et al. 2017	21	The one describing the association between SES and caries increment with OR (CI) and one of the latest.
Brazilian schoolchildren from Piracicaba, SP	Tagliaferro EP et al. 2006 [61]<br>Tagliaferro EP et al. 2008 [62]	Tagliaferro EP et al. 2006	18	The one with the representative population.
Study from Urban region of Manaus, Brazil	Costa, LR et al. 2023 [63]<br>Gomes AC et al. 2023 [64]<br>Monteiro MCC et al. 2025. [65]	Costa, LR et al. 2023	4	The one with estimated CI. Also describes the line between SES and caries incidence.
Study from Foshan city in Guangdong Province in southern China	Wang K et al. 2020 [66]<br>Pang L et al. 2021 [67]	Wang K et al. 2020	20	The one with the highest sample size.
Singapore Dental Council	Tan SHX et al. 2021 [68]<br>Tan SHX et al. 2024 [69]	Tan SHX et al. 2021	19	Directly focusing on the association between factors in childhood and caries incidence.

References for Appendix 1. 5 and Appendix 1. 6.

[1] Alves FN, de Andrade CL, Vettore MV. Planning oral health care using the sociodental approach and the index of family living conditions: a cross-sectional study in Brazilian adolescents. BMC Res Notes. 2015;8:588.

[2] Birkeland JM, Haugejorden O, von der Fehr FR. Analyses of the caries decline and incidence among Norwegian adolescents 1985-2000. Acta Odontol Scand. 2002;60:281-9.

[3] Brusius CD, Alves LS, Maltz M. Is patient's caries activity associated with caries increment among adolescents, regardless of caries experience? Caries Res. 2023; 2023;57: 613-18.

[4] Borowska-Struginska B, Zadzinska E, Bruzda-Zwiech A, Filipinska R, Lubowiecka-Gontarek B, Szydlowska-Walendowska B, et al. Prenatal and familial factors of caries in first permanent molars in schoolchildren living in urban area of Lodz, Poland. Homo. 2016;67:226-34.

[5] Broadbent JM, Zeng J, Foster Page LA, Baker SR, Ramrakha S, Thomson WM. Oral Health-related Beliefs, Behaviors, and Outcomes through the Life Course. J Dent Res. 2016;95:808-13.

[6] Brondani B, Knorst JK, Emmanuelli B, Gasperini MRC, Braga MM, Ardenghi TM, et al. Do progression rates of initial and moderate caries lesions and sound surfaces of primary teeth increase significantly after 7 years? Int J Paediatr Dent. 2025;35:80-9.

[7] Carbajal Rodriguez G, van Meijeren-van Lunteren AW, Wolvius EB, Kragt L. Poverty Dynamics and Caries Status in Young Adolescents. Community Dent Oral Epidemiol. 2025;53:90-7.

[8] Cianetti S, Abraha I, Pagano S, Lupatelli E, Lombardo G. Sonic and ultrasonic oscillating devices for the management of pain and dental fear in children or adolescents that require caries removal: a systematic review. BMJ Open. 2018;8 (4):e020840.

[9] Cianetti S, Lombardo G, Lupatelli E, Rossi G, Abraha I, Pagano S, et al. Dental caries, parents' educational level, family income and dental service attendance among children in Italy. Eur J Paediatr. Dent 2017;18:15-8.

[10] David J, Raadal M, Wang NJ, Strand GV. Caries increment and prediction from 12 to 18 years of age: a follow-up study. Eur Arch Paediatr Dent. 2006;7:31-7.

[11] Ditmyer MM, Mobley C, Draper Q, Demopoulos C, Smith ES. Development of a theoretical screening tool to assess caries risk in Nevada youth. J Public Health Dent. 2008;68:201-8.

[12] Ditmyer M, Dounis G, Mobley C, Schwarz E. Inequalities of caries experience in Nevada youth expressed by DMFT index vs. Significant Caries Index (SiC) over time. BMC Oral Health. 2011;11:12.

[13] Divaris K, Fisher EL, Shugars DA, White RP, Jr. Risk factors for third molar occlusal caries: a longitudinal clinical investigation. J Oral Maxillofac Surg 2012;70:1771-80.

[14] Ghazal TS, Levy SM, Childers NK, Carter KD, Caplan DJ, Warren JJ, et al. Survival analysis of caries incidence in African-American school-aged children. J Public Health Dent. 2019;79:10-7.

[15] Goncalves BML, Armada L, Stea TH, Rebelo MAB, Carvalho JC, Goncalves LS, et al. Bidirectional association between dental caries and body mass index in socially vulnerable adolescents: a group-based trajectory analysis. J Dent. 2025;162:106087.

[16] Haugejorden O, Magne Birkeland J. Ecological time-trend analysis of caries experience at 12 years of age and caries incidence from age 12 to 18 years: Norway 1985-2004. Acta Odontol Scand. 2006;64:368-75.

[17] Holmen A, Stromberg U, Magnusson K, Twetman S. Tobacco use and caries risk among adolescents--a longitudinal study in Sweden. BMC Oral Health. 2013;13:31.

[18] Holst D, Schuller AA. Oral health in a life-course: birth-cohorts from 1929 to 2006 in Norway. Community Dent Health. 2012;29:134-43.

[19] Hummel R, van der Sanden W, Bruers J, van der Heijden G. The relationship between claimed restorations and future restorations in children and adolescents: An observational follow-up study on risk categories for dental caries. PLoS One. 2021;16:e0259495.

[20] Khoramrooz M, Mirrezaie SM, Emamian MH, Dadgari A, Hashemi H, Fotouhi A. Decomposition of economic inequalities in dental caries among Iranian schoolchildren. PLoS One. 2024;19:e0306778.

[21] Liew L, Saipollah F, Hassan A, MZ. R. The association of sociodemographic factors and dental caries among secondary school children in Jempol district of Negeri Sembilan. JUMMEC. 2024;27 (2).

[22] Lewsey JD, Thomson WM. The utility of the zero-inflated Poisson and zero-inflated negative binomial models: a case study of cross-sectional and longitudinal DMF data examining the effect of socio-economic status. Community Dent Oral Epidemiol. 2004;32:183-9.

[23] Lieske B, Schenk L, Kuhr K, Pitchika V, Borof K, Jordan AR, et al. Association between migration history and oral health: results of the 6th German Oral Health Study (DMS * 6). Quintessence Int. 2025;56:S126-S34.

[24] Masood M, Yusof N, Hassan MI, Jaafar N. Longitudinal study of dental caries increment in Malaysian school children: a 5-year cohort study. Asia Pac J Public Health. 2014;26:260-7.
[25] Meira GF, Knorst JK, Maroneze MC, Ortiz FR, Ardenghi TM. Effect of dental caries and socioeconomic status on social capital throughout adolescence: a 6-year follow-up. Braz Oral Res. 2020;34:e104.
[26] Meyer K, Geurtsen W, Gunay H. An early oral health care program starting during pregnancy: results of a prospective clinical long-term study. Clin Oral Investig. 2010;14:257-64.
[27] Nicolau B, Marcenes W, Bartley M, Sheiham A. Associations between socio-economic circumstances at two stages of life and adolescents' oral health status. J Public Health Dent. 2005;65:14-20.
[28] Nishioka D, Saito J, Ueno K, Kondo N. Single-parenthood and health conditions among children receiving public assistance in Japan: a cohort study. BMC Pediatr. 2021;21:214.
[29] Noro LR, Roncalli AG, Teixeira AK. Contribution of cohort studies in the analysis of oral health in children and adolescents in Sobral, Ceará. Rev Bras Epidemiol. 2015;18:716-9.
[30] Noronha TG, Knorst JK, da Silveira Borstman E, Ardenghi T, Tomazoni F. Adverse childhood experiences and dental caries among adolescents. Caries Res. 2025;1-8.
[31] Ogwo C, Brown G, Warren J, Caplan D, Levy S. Dental caries incidence and associated factors in young adults. J Public Health Dent. 2023;83:347-54.
[32] Östberg AL, Petzold M. A longitudinal study of the impact of change in socioeconomic status on dental caries in the permanent dentition of Swedish children and adolescents. Community Dent Oral Epidemiol. 2020;48:271-9.
[33] Peres KG, Peres MA, Araujo CL, Menezes AM, Hallal PC. Social and dental status along the life course and oral health impacts in adolescents: a population-based birth cohort. Health Qual Life Outcomes. 2009;7:95.
[34] Peres MA, Peres KG, de Barros AJ, Victora CG. The relation between family socioeconomic trajectories from childhood to adolescence and dental caries and associated oral behaviours. J Epidemiol Community Health. 2007;61:141-5.
[35] Peres KG, Peres MA, Demarco FF, Tarquinio SB, Horta BL, Gigante DP. Oral health studies in the 1982 Pelotas (Brazil) birth cohort: methodology and principal results at 15 and 24 years of age. Cad Saúde Pública. 2011;27:1569-80.
[36] Ruiz B, Broadbent JM, Thomson WM, Ramrakha S, Hong CL, Poulton R. Differential Unmet Needs and Experience of Restorative Dental Care in Trajectories of Dental Caries Experience: A Birth Cohort Study. Caries Res. 2023;57:524-35.
[37] Schmidt J, Vogel M, Poulain T, Kiess W, Hirsch C, Ziebolz D, et al. Association of Oral Health Conditions in Adolescents with Social Factors and Obesity. Int J Environ Res Public Health. 2022;19(5).
[38] Sousa FS, Lopes BC, Costa EM, Alves CMC, Queiroz RCS, Tonello AS, et al. Do social inequalities persist in the distribution of dental caries in adolescents from Maranhao? Contributions of a population-based study. Cien Saude Colet. 2021;26:2625-34.
[39] Stangvaltaite L, Kundzina R, Bolstad NL, Eriksen HM, Kerosuo E. Deep carious lesions and other consequences of caries among 18-year-olds at Public Dental Health Service in Northern Norway: A cross-sectional age cohort study. Acta Odontol Scand. 2015;73:401-7.
[40] Swedberg Y, Noren JG. A 15-year series analysis of caries development related to a socioeconomic variable. A study of the year group leaving the organised dental care in Goteborg, Sweden. Swed Dent J. 2003;27:151-8.
[41] Taani DQ. Relationship of socioeconomic background to oral hygiene, gingival status, and dental caries in children. Quintessence Int. 2002;33:195-8.
[42] Teixeira AK, Roncalli AG, Noro LR. Factors related to the dental caries incidence in youth: a cohort study in Brazilian Northeastern. Cien Saude Colet. 2016;21:3871-8.
[43] Teixeira AKM, Roncalli AG, Noro LRA. Income Trajectories and Oral Health of Young People in a Life Course Study. Caries Res. 2019;53:347-56.
[44] Thomson WM, Poulton R, Kruger E, Boyd D. Socio-economic and behavioural risk factors for tooth loss from age 18 to 26 among participants in the Dunedin Multidisciplinary Health and Development Study. Caries Res. 2000;34:361-6.
[45] Virtanen JI. Changes and trends in attack distributions and progression of dental caries in three age cohorts in Finland. J Epidemiol Biostat. 2001;6:325-9.
[46] Ying Y, Nicolau B, Van Hulst A, Henderson M, Madathil S, Barnett T: Socio-environmental typologies and incidence of dental caries from childhood to adolescence among youth in Montreal: The QUALITY Cohort. Caries Res. 2025; Jun 9:1-10. doi: 10.1159/000546747.
[47] Alm A, Wendt LK, Koch G, Birkhed D, Nilsson M. Caries in adolescence - influence from early childhood. Community Dent Oral Epidemiol. 2012;40:125-33.

[48] Alm A, Wendt L, Koch G, Birkhed D. Prevalence of approximal caries in posterior teeth in 15-year-old Swedish teenagers in relation to their caries experience at 3 years of age. Caries Res. 2007;41(5):392-8.
[49] Källestål C, Fjelddahl A. A four-year cohort study of caries and its risk factors in adolescents with high and low risk at baseline. Swed Dent J. 2007;31:11-25.
[50] Källestål C, Wall S. Socio-economic effect on caries. Incidence data among Swedish 12-14-year-olds. Community Dent Oral Epidemiol. 2002;30:108-14.
[51] Peres MA, Liu P, Demarco FF, Silva AER, Wehrmeister FC, Menezes AM, et al. Income trajectories affect treatment of dental caries from childhood to young adulthood: a birth cohort study. Braz Oral Res. 2018;32:e36.
[52] Peres MA, Barros AJ, Peres KG, Araujo CL, Menezes AM. Life course dental caries determinants and predictors in children aged 12 years: a population-based birth cohort. Community Dent Oral Epidemiol. 2009;37:123-33.
[53] Ortiz FR, Reyes LT, Ardenghi TM. Social Economic Disadvantage and Untreated Dental Caries: Findings from a Cohort Study in Adolescents. Caries Res. 2022;56:179-86.
[54] Ortiz AS, Tomazoni F, Knorst JK, Ardenghi TM. Influence of socioeconomic inequalities on levels of dental caries in adolescents: A cohort study. Int J Paediatr Dent. 2020;30:42-9.
[55] Brondani B, Knorst JK, Ardenghi TM, Mendes FM, Brondani MA. Community and individual socioeconomic inequalities and dental caries from childhood to adolescence: A 10-year cohort study. Community Dent Oral Epidemiol. 2024;52:540-9.
[56] Knorst JK, Brondani B, Vettore MV, Hesse D, Mendes FM, Ardenghi TM. Pathways between Social Capital and Oral Health from Childhood to Adolescence. J Dent Res. 2022;101:1155-64.
[57] Wang X, Bernabe E, Pitts N, Zheng S, Gallagher JE. Dental Caries Clusters among adolescents in England, Wales, and Northern Ireland in 2013: implications for proportionate universalism. Caries Res. 2021;55(6):563-76.
[58] Curtis AM, Cavanaugh JE, Levy SM, VanBuren J, Marshall TA, Warren JJ. Examining caries aetiology in adolescence with structural equation modelling. Community Dent Oral Epidemiol. 2018;46:258-64.
[59] Chankanka O, Cavanaugh J, Levy S, Marshall T, Warren J, Broffitt B, et al. Longitudinal associations between children's dental caries and risk factors. J Public Health Dent. 2011; 71:289-300.
[60] Broffitt B, Levy SM, Warren J, Cavanaugh JE. Factors associated with surface-level caries incidence in children aged 9 to 13: the Iowa Fluoride Study. J Public Health Dent. 2013;73:304-10.
[61] Tagliaferro EP, Pereira AC, Meneghim Mde C, Ambrosano GM. Assessment of dental caries predictors in a seven-year longitudinal study. J Public Health Dent. 2006;66:169-73.
[62] Tagliaferro EP, Ambrosano GM, Meneghim Mde C, Pereira AC. Risk indicators and risk predictors of dental caries in schoolchildren. J Appl Oral Sci. 2008;16:408-13.
[63] Costa LR, Vettore MV, Quadros LN, Rebelo Vieira JM, Herkrath A, Queiroz AC, et al. Socio-economic status, psychosocial factors, health behaviours and incidence of dental caries in 12-year-old children living in deprived communities in Manaus, Brazil. J Dent. 2023;133:104504.
[64] Gomes AC, Vettore MV, Quadros LN, Rebelo MAB, Rebelo Vieira JM. Does using the sociodental approach in oral health care influence use of dental services and oral health of adolescents living in deprived communities? A one-year follow-up study. BMC Health Serv Res. 2023;23:605.
[65] Monteiro MCC, Rebelo MAB, de Freitas YNL, Rebelo Vieira JM, Vettore MV. The Influence of Social Support, Oral Health Beliefs, and Health Behaviours on Dental Caries in Children Living in Deprived Neighbourhoods. Caries Res. 2025;59:228-36.
[66] Wang K, Pang L, Fan C, Cui T, Yu L, Lin H. Enamel and Dentin Caries Risk Factors of Adolescents in the Context of the International Caries Detection and Assessment System (ICDAS): A Longitudinal Study. Front Pediatr. 2020;8:419.
[67] Pang L, Wang K, Tao Y, Zhi Q, Zhang J, Lin H. A New Model for Caries Risk Prediction in Teenagers Using a Machine Learning Algorithm Based on Environmental and Genetic Factors. Front Genet. 2021;12:636867.
[68] Tan SHX, Teo YY, Tan MHX, Gao X. Childhood Factors and Dental Caries in the Permanent Dentition: Findings of an 8-Year Study Under a Nationwide School Dental Service. Int Dent J. 2021;71:508-15.
[69] Tan SHX, Teo YY, Tan MHX, Heng WY, Chen W, Gao X. Caries trajectories from childhood to adolescence: Analysis of data from a nationwide school dental service. Community Dent Oral Epidemiol. 2024;52:900-10.

Appendix 1. 7. Definitions and integration methods of composite SES.

First author (Study ID)	*Definitions used*	*Integration methods*
Brondani et al. (3)	Individual SES was evaluated by monthly household income, maternal and paternal education, and household crowding.	*Monthly household income:* 4 quartiles from lowest (Q1) to Highest (Q4) according to Reais (Brazilian currency). *Parental education:* formal education <8 years or ≥ 8 years. *Household crowding:* 4 quartiles from lowest (Q1) to Highest (Q4) according to people per room.
Costa et al. (4)	SES variable encompassed the number of goods, house overcrowding, parents' schooling, and monthly family income.	*Number of durable goods at home:* according to a list of 11 items. *Overcrowded accommodation* (dividing the number of residents by the number of rooms): Values: 0 to 1.99, 2 to 2.99, 3 or more. *Parents' education:* 1 to 7 years, 8 to 11 years, 12 years or more. *Family income (monthly)* in Brazilian minimal wages (BMW): ≤ ½ BMW, ½ to 1 BMW, > 1 BMW
Page et al. (13)	SES was recorded by one area-based measure and by one household measure.	*The area-based measure:* New Zealand Deprivation 2001 census data index, which combines nine variables reflecting both social and material deprivation. Also, geocoding of each participant's street address was done to match the areas. *The household measure* used, is is based on parents' occupation. The highest SES of the parents was used.
Hall-Schullin et al. (6)	SES according to the Index for Deprivation (Index of Material Deprivation quintiles (IMDQ)).	*IMDQ*: combines information from 7 domains, including income, employment, crime, and health. IMDQ 1 (least deprived), IMDQ 2, IMDQ 3, IMDQ 4, IMDQ 5 (most deprived).
Gerdin et al. (5)	SES. Five distinct clusters with homogeneous socioeconomic characteristics were made.	*Socioeconomic clusters:* based on available information on available income, social allowances, and parental educational level, categorized as I (high), II, III, IV, and V (low).
Jung et al. (8)	SES according to the Family Affluence Scale (FAS).	*FAS scores* (0-9), High: >5, Low: ≤ 5. The scores were calculated by summing up dummy variables to represent the ownership of a family car, a private bedroom for the student, computers, and the number of family vacations in the past year.
